# OncoCardioDB: a public and curated database of molecular information in onco-cardiology/cardio-oncology

**DOI:** 10.1093/database/baad029

**Published:** 2023-05-09

**Authors:** Angela L Riffo-Campos, Juan Domingo, Esther Dura

**Affiliations:** Millennium Nucleus on Sociomedicine (SocioMed), Universidad de La Frontera, Doctorado en Ciencias Médicas, C/Francisco Salazar, 1145 Temuco, Chile; Department of Computer Science, ETSE, Univ. de Valencia, Avda. de la Universidad, s/n, Burjasot, 46100 Valencia, Spain; Department of Computer Science, ETSE, Univ. de Valencia, Avda. de la Universidad, s/n, Burjasot, 46100 Valencia, Spain; Department of Computer Science, ETSE, Univ. de Valencia, Avda. de la Universidad, s/n, Burjasot, 46100 Valencia, Spain

## Abstract

Numerous studies have been published which, separately, investigate the influence of molecular features on oncological and cardiac pathologies. Nevertheless, the relationship between both families of diseases at the molecular level is an emerging area within onco-cardiology/cardio-oncology. This paper presents a new open-source database that aims to organize the curated information concerning the molecular features validated in patients involved in both cancer and cardiovascular diseases. Entities like gene, variation, drug, study and others are modelled as objects of a database which is populated with curated information from 83 papers identified by systematic literature searched for up to 2021. Researchers will discover new connections among them to validate hypotheses or suggest new ones. Special care has been taken to use standard nomenclature for genes, pathologies and all the objects for which accepted conventions exist. The database can be consulted via the web with a system of simplified queries, but it also accepts any query. It will be updated and refined with the incorporation of new studies as they become available.

**Database URL** http://biodb.uv.es/oncocardio/

## Introduction

Onco-cardiology/cardio-oncology studies the relationship between cardiovascular diseases (CVDs) and cancer (1). It is well known that these families of diseases have a high incidence in the world’s population. Indeed, among non-communicable diseases, CVDs are the first cause of death (2), whereas cancer is second (3). CVDs and cancer historically have been studied separately, but the growing clinical evidence in favour of the relationship between them gave rise to onco-cardiology field in 2010 (1). These relationships can be approached in two ways: risk factors shared by both diseases and cardiotoxicity derived from cancer treatments. Common risk factors include smoking, physical inactivity, genetic predisposition and advanced age, among others (4). Cardiotoxicity derived from cancer treatment includes all types of cancer and therapy (5–7), and it is recognized as one of the main causes of mortality among cancer survivors (8), especially with regard to breast cancer (9). Cardiotoxicity impacts the prognosis of treated patients in both the short and long terms; indeed, it is possible to develop CVDs derived from cancer treatment years after having overcome the disease (10). Emerging evidence suggests that these diseases share molecular pathways such as chronic inflammation, oxidative stress, aberrant apoptosis and angiogenesis (11). Other studies have identified common molecular targets for both diseases, including the widely used cardiac biomarkers troponins and natriuretic peptides (12). Furthermore, evidence (publications and citations) related to cardio-oncology research increased almost exponentially from 2010 to date (13).

From the former consideration, it seems important to store and organize all the molecular information generated so far in the emerging field of onco-cardiology. This would allow the assimilation of all the information related to molecular relationships between CVDs and cancer in a timely manner. It would also be useful in research and clinical practice, since in the long term, it will contribute to identifying new and safer preventive and therapeutic approaches for both diseases. This implies certain challenges, one of them being the differences in the way of expressing onco-cardiology information. Such differences suggest that some effort should be made to represent and store this information in a suitable, common way. This way must be simultaneously well standardized, well organized around the key concepts and fully accessible in order to extract valuable knowledge from it.

Several databases exist that store, organize and make available the information, on the one hand, concerning cancer and, on the other hand, related to CVDs and molecular factors associated with pathologies in general. Some reviews have been published regarding the cancer databases (14), e.g. The Cancer Genome Atlas (TCGA). The TCGA is a research network that stores and analyses a large number of human tumours to discover molecular aberrations at the Deoxyribonucleic acid (DNA), Ribonucleic acid (RNA), protein and epigenetic levels, with the aim of improving our ability to diagnose, treat and prevent cancer. The data are accessible through the Genomic Data Commons (GDC) Data Portal (15) (accessible at https://portal.gdc.cancer.gov/). The GDC Data Portal is a robust platform that allows you to search and download omics and clinical cancer data for analysis and includes 70 projects on 67 primary cancer sites with >85 0000 cases.

With respect to CVDs, the CVD (16) and CardioGenBase (17) databases have been created to store and organize information related to multi-omic studies. Regarding the molecular bases of the disease, in general, we can mention the Online Mendelian Inheritance in Man (OMIM). The OMIM (available at https://www.omim.org) contains comprehensive, authoritative and timely information concerning human genes and genetic disorders, obtained from biomedical literature, focusing on the relationship between the phenotype and genotype. It aims to support human genetics research and education and the practice of clinical genetics (18).

Regarding efforts to store information related to onco-cardiology, the Virtual Cardio-Oncology Research Initiative aims to create a large-scale resource, with a long follow-up period, to provide quality data for research and identify new scientific avenues to further knowledge of cardio-oncology (19). The importance of big data in the area of cardio-oncology has also been highlighted (20). However, to the best of our knowledge, there is no database for molecular features specifically involved in onco-cardiology/cardio-oncology. Due to all of this, the proposal and main goal of this paper is to create a database whose entities are the key concepts used in studies concerning onco-cardiology and whose relationship reflects the real molecular interactions between them. Apart from the database itself, mechanisms should be provided to allow easy interaction with it by biomedical practitioners. This motivates the need to implement a user-friendly interface and to provide complete accessibility through the Internet at all times.

## Materials and methods

### Overview

**Figure 1.**
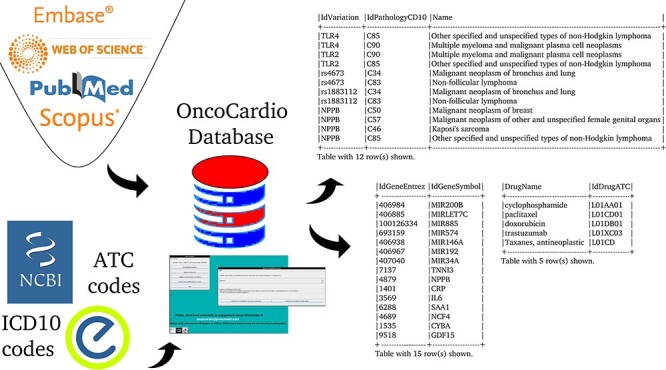
An overview of the information contained in the database.

The aim is to create a database that organizes and makes available concepts and knowledge related to molecular information on onco-cardiology revealed by human studies which have been reported in original articles published to date (Figure 1).

The first step to accomplish this objective was the design of the database itself; namely, which concepts appear in all studies, what their data types and allowed values are and how they are related to each other. This information was used to choose the entities and relationships of the database.

The second step involves human intervention in the form of a knowledgeable reader who can identify in each medical paper the chosen concepts (see part 1 of Supplementary material to access the referenced papers) and take note of them as fields in a key-value table. The said table will be used to feed the database. Possible errors in curation, especially those related to typing mistakes or the use of nonstandard terms and/or invalid values, should be, and were, automatically reported and corrected before the acceptance of the data using text analysis programs.

The third step was to identify which queries are interesting and to write them in a formal language, structured query language (SQL) in this case. For users not familiar with SQL, a number of ‘predefined queries’, were provided. Also, a graphical representation of the diagram of tables is provided to allow expert users to write ‘advanced queries’ (any arbitrary query) in SQL. It is important to point out that few biological databases allow such unrestricted access. In any case, the users can contact us if their question is not covered and we will include a predefined query that answers it.

The last step was to build a simple user-friendly graphical interface for both the predefined and advanced queries, which would allow the user to access through the Internet, make his/her queries and obtain the results. The results must be provided in a form suitable for two purposes: automatic processing by machines and visual human-understandable representation. The formats chosen for that were CSV (comma-separated values to be read by a spreadsheet) and HTML (Hypertext Markup Language to be shown in a normal web browser), respectively.

### Data collection

The data correspond to all the molecular target information that exists to date on the relationship between cancer and CVDs that has been validated in patients over 18 years of age. The strategy used to obtain the primary information was a systematic search of the literature, according to the PRISMA statement, using four search strategies in Medline, Web of Science, Scopus and Embase, which are detailed in the table at the end of part 1 of Supplementary material, ‘References of the papers used to populate the OncoCardio database with their accessible URLs and search strategies used.’

The inclusion criteria of the articles were original publications, scientific research and clinical studies focused on the molecular relationship between cancer and CVDs in adult patients (>18 years of age). Those studies that do not include molecular features (protein, DNA, RNA, methylation, etc.) or those in which the molecular features were evaluated in animal models, cell lines, and xenograft, among others, not validated directly in human samples, were excluded. Non-original research, grey literature, clinical trial protocols, reviews, consensus documents, short communications, opinions papers, letters, posters and conference abstracts were also excluded. A subselection of this information was published in a scoping review (see (22)). In that previous work, exploratory research was carried out on the available evidence on novel molecular biomarkers associated with cardiotoxicity in the adult population undergoing cancer therapy. A total of 42 studies were included, and the data were collected in a spreadsheet and summarized in the publication. In the present work, the relationship between cancer and CVDs was studied in a broader sense (not only under cardiotoxicity), and the information was stored in a database. Therefore, the information contained in the selected articles was curated, putting in consensus the names of the genes, including their genomics position, an official symbol for human, synonyms, official identifiers, drug codes and pathology codes. This data curation was carried out by expert judgement, and if the information could not be normalized, the paper was discarded. Of the 91 selected papers, 7 were discarded for this reason, while articles that studied metabolites, lipids and other molecules that are not possible to assign easily/directly to an area of the genome were discarded in the full-text reading phase.

### Database organization

**Figure 2.**
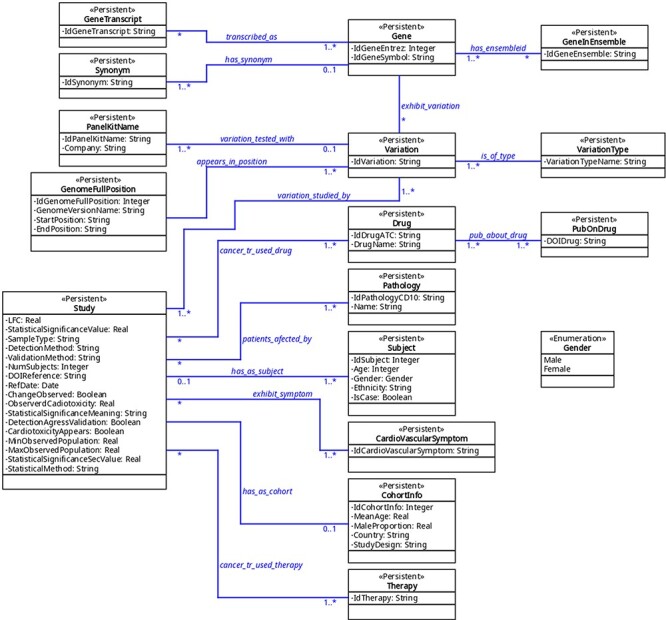
The UML diagram of the database.

The organization of the database was designed using variation as a key concept; variation in this context means ‘a change in amount or level’ or ‘a change of organization or arrangement’. This is because these are the molecular units on which there can be a molecular relationship between cancer and CVDs. This ‘Variation’ object can be a finite number of types (‘VariationType’), which refers to its role in biological processes. Currently, 10 types are admitted, represented in the database under the same name: SNV (single-nucleotide variant), SNP (single-nucleotide polymorphism), InDel (insertion and deletion), CNV (copy number variation), abundance proteins, differential expression, DNA methylation, RNA methylation, histone modifications and post-translational modification. Others can be added if needed. This variation, in turn, may or may not belong to a gene (object ‘Gene’); when it does not, it is intergenic and maps to the closest gene. The gene is identified by its identifiers (namely, Entrez and Ensembl), synonyms, and transcripts. On the other hand, a variation has a specific position in the genome (object ‘GenomeFullPosition’). Also, it may be used as a biomarker in a panel (object ‘PanelKitName’). Furthermore, this variation must have been reported as related to cancer and CVDs in at least one reported study (object ‘Study’). This study has specific fields that define the clinical context in which the variation is associated with both pathologies, including statistics values, abundance (e.g. fold change) and phenotype characteristics (see Supplementary material ‘Tables corresponding to entities’ and Figure 2). The relationship between these diseases can be approached from the risk factors that cancer and CVDs have in common, from the possibility that one can be a risk factor for the other one and from the cardiotoxicity that cancer therapies produced in the patients. This is addressed in the database using a combination of fields such as ‘CarditoxicityAppears’ (which can be null when corresponding to another type of association), ‘IdPathologyCD10’ (which can refer to cancer or CVDs) or IdCardiovascularSymptom (to describe the symptoms). See part 2 of Supplementary material for a full description.

### Knowledge extraction and curation

To fill the database with the information extracted from the medical literature, the biomedical people read the papers and fill in a form for each one (in our case simply as cells on a spreadsheet) which is exported in text format and read by programs that process it and automatically generate the SQL sentences. The said programs were written in Perl (Practical Extraction and Report Language).

The values of the numerous fields are checked first to verify that they are of the expected type (i.e. strings of characters, integer numbers and floating point numbers in a certain range) and also to be sure that they take a legal value, i.e. if they belong to a finite set, the value is one of its elements. This is done, e.g., to check for the validity of the code of a drug or of a pathology and so on.

Furthermore, very large sets of values must be incorporated into the tables. For instance, the table ‘Gene’ contains the Entrez identifier of every gene in the human genome. The same thing occurs with every possible drug classified by the Anatomical Therapeutic Chemical Classification (ATC) (23), and every disease included in the International Classification of Diseases, ver. 10 (ICD10) (24). It can be argued that only a small fraction of all the genes, drugs or diseases will appear in the field of onco-cardiology, but we have decided to incorporate the whole list. This has not been a problem since this incorporation was done automatically with Perl macros and the size of the complete database turned out to be well below the capacity manageable by current computers. Moreover, in this way the filled tables can be useful for databases in other biomedical areas.

Curation of manually extracted data by automatic or semi-automatic means is relatively common in these kinds of applications; other systems that mention this or similar approaches are Datanator (25) or MarkerDB (21). Notice that special care has been taken to use reliable sources of information and standardized lists, such as the ATC or ICD10 (see section 1 of part 2 of Supplementary material).

### Software architecture of the system

The whole system was composed at the software level by an http microserver written in JavaScript and running with node.js. Users can provide an email address that is not permanently stored and is used exclusively to send the results of the queries to the user in CSV and/or HTML. Nevertheless, providing an email address is not compulsory: a user can log in anonymously simply to test, interact with the database and visualize and download the results. The database server runs in MariaDB. Database internal users have been created which have read-only access to the database tables, so even with access to an SQL console, they cannot alter its integrity. A program written in Java functions as the graphical user interface (GUI) to the database and it is accessed through a Guacamole server (26) that opens a session for each user. The user can also access the web of the ATC and the ICD10 to consult drug or pathology codes. A daemon written in Perl orchestrates the former pieces, calling them to be run whenever needed.

The system is currently fully functional and can be found at https://biodb.uv.es/oncocardio.

## Results

### Graphical interface and remote access design

The tasks the user can execute are the following:

- Perform a predefined query whose input is introduced through a form and whose result is shown on the screen and also temporarily stored to be sent by an email or downloaded at the end of the session.
- Perform an advanced query, which is freely written in SQL into a text input area. The results are shown and stored, in the same way as it was done for predefined queries.
- Write his/her query in plain English that will be sent to us, as explained in the section ‘Description and examples of predefined queries’.
- Report the bibliographic reference of a new publication in this field whose results their authors would like to see incorporated into OncoCardio.
- Leave the session. At that point, the user is requested to choose the format or formats (CSV, HTML or both) for the results to be either sent by an email or downloaded in the case of anonymous log-in.

Additionally and at any time, the user can get help through a menu at the upper side of each window in the form of a text and graphical diagram of the tables and fields in the database. Also, he/she can connect to the websites of ATC (23) and ICD10 (24) to find or verify the drug and disease codes, respectively. A complete demonstration of the use of the system is shown in the video included as Supplementary material.

### Database statistics

The statistics information has been directly obtained from the data base (DB) itself through appropriate SQL queries (see part 3 of Supplementary material for full statistics).

**Figure 3.**
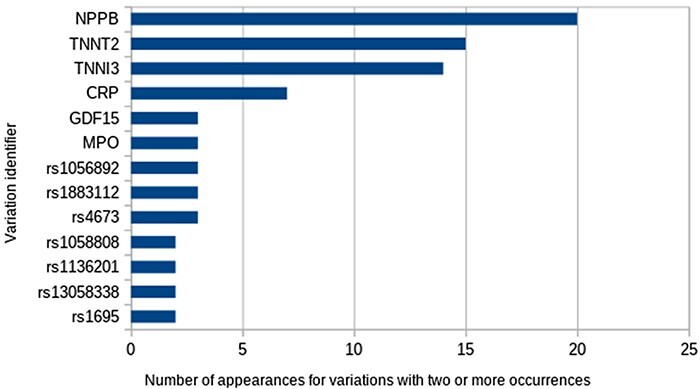
The most frequent variations.

A total of 93 unique Entrez identifiers were reported from all 61 548 possible identifiers. The most frequent were Entrez 4879 (*NPPB*), 7139 (*TNNT2*) and 7137 (*TNNI3*), but most identifiers appeared just once. With respect to variations, 107 different variations appear in at least one study. The most frequent ones were variations in the abundance of proteins encoded by the genes already mentioned (Figure 3). Regarding the types of variations, all those reported to date fall into the categories of SNVs, differential expression and variation in protein abundance, being studied mainly in blood samples.

The total number of diseases stored with their corresponding ICD10 codes is 46 654. Out of them, 23 are reported in at least one study. Their ordering by the number of occurrences shows that the most frequent pathology is a malignant neoplasm of breast (ICD10 code: C50), which appears 122 times (Table 3).

**Table 3.** Top five most frequent types of cancer reported to date in the OncoCardio database

CD10	Name	Frequency
C50	Malignant neoplasm of breast	122
C85	Other specified and unspecified types of non-Hodgkin lymphoma	24
C34	Malignant neoplasm of bronchus and lung	22
C95	Leukaemia of unspecified cell type	12
C90	Multiple myeloma and malignant plasma cell neoplasms	11

Six different basic therapies (chemotherapy, hormonal therapy, immunotherapy, radiotherapy, surgery and targeted therapy) have been considered since no others are mentioned in any study, and also any combination of two or more of them is in principle possible, which generates 64 possibilities. From these 64, only 7 different possibilities appear in the studies. The most frequent is chemotherapy alone, which was used 144 times, followed by radiotherapy with 39 occurrences. The most common combined therapy is chemotherapy+radiotherapy in the fourth place with 15 occurrences.

The total number of drugs stored in the DB with their corresponding ATC code is 6460. Out of them, 259 were used in any study. This number is greater than the number of studies since the prescribed treatment in many studies uses two or more drugs. In fact, only 26 of them are different. The most frequently used drug is doxorubicin (ATC code: L01DB01) with 58 occurrences, followed by cyclophosphamide (ATC code: L01AA01) with 35 occurrences. Finally, there are eight drugs mentioned in only one study each.

The total number of different cardiovascular symptoms reported in the studies is 42. Ordering by the frequency of appearance shows that the decline in left ventricular ejection fraction (LVEF) is the most common with 125 occurrences, followed by heart failure with 20 occurrences. On the other hand, 15 symptoms are mentioned only once each.

### Description and examples of predefined queries

The database contains a collection of (currently) seven predefined searches (Queries 1–7) that answer common questions. Among them, only one field is left free and the user is requested to provide a value. The predefined queries are as follows (values in typewriter font correspond to their denomination in the tables (see Figure 2 and part 3 of Supplementary material).

1. Input: a pathology code (IdPathologyCD10).
  Output: all variations (as IdVariation) associated to the input pathology and, for each of these variations, all its associated pathologies.
2. Input: a variation name (IdVariation).
  Output: all pathologies (as IdPathologyCD10) associated with that variation and, for each of them, all studies (as DOIReference) and, for each study, all symptoms (if any) associated with it (as IdCardiovascularSymptom).
3. Input: a variation (IdVariation).
  Output: all panel kits (as IdPanelKitName) that can test the expression of the variation.
4. Input: a variation (IdVariation).
  Output: all drugs (as IdDrugATC) which have been used with patients that exhibit the variation.
5. Input: a pathology name (IdPathologyCD10).
  Output: all variations (as IdVariation) detected in any study associated with that pathology and, for each of them, the gene (as IdGeneSymbol) in which that variation occurs and the number of studies that refer to the found association.
6. Input: a gene (IdGeneSymbol).
  Output: all variations (as IdVariation) that occur in that gene and, for each of them, all the drugs (as IdDrugATC) that are supposed to modify the variation.
7. Input: a gene (IdGeneSymbol).
  Output: all variations (as IdVariation) that occur in that gene and, for each of them, all the panel kits (as IdPanelKitName) that can test the expression of that variation.

For example, the user might want to know what onco-cardiology genes have been identified in lung cancer and how many studies have reported said association? This can be answered using Query 5, giving, as input the ICD10-code of the pathology. In this example, we use C34 (malignant neoplasm of bronchus and lung). The result is Table 1, which shows all the reported genes and the number of studies.

**Table 1.** Genes related to oncocardiology in malignant neoplasm of bronchus and lung (ICD10 code: C34) and the number of studies that report the association

ID gene Entrez	Symbol	COUNT(DISTINCT s.IdStudy)
1401	CRP	3
1535	CYBA	1
3569	IL6	1
4689	NCF4	1
4879	NPPB	3
6288	SAA1	1
7137	TNNI3	4
9518	GDF15	1
406 885	MIRLET7C	1
406 938	MIR146A	1
406 967	MIR192	1
406 984	MIR200B	1
407 040	MIR34A	1
693 159	MIR574	1
100 126 334	MIR885	1

Compound questions are also included, such as which pathology is associated with a molecular feature, which cardiovascular symptoms are associated with it and which papers report said association? This can be answered in Query 2. The input in this case is a variation; for example, let us use myeloperoxidase (MPO). MPO was always reported in breast cancer but associated with three cardiovascular symptoms in two different studies, as shown in Table 2.

**Table 2.** Results obtained using Query 2, with MPO variation as input

Pathology name	Cardiovascular symptom	DOIReference
Malignant neoplasm of breast	Cancer therapy–related cardiac dysfunction	10.1161/JAHA.119.014708
Malignant neoplasm of breast	Decline in LVEF	10.1373/clinchem.2015.241232
Malignant neoplasm of breast	Heart failure	10.1373/clinchem.2015.241232

The list of predefined queries is obviously not exhaustive and can be augmented in the future if the authors receive feedback from the users concerning what they would like to get from the database. To do so, the program provides a specific item where the users can write their queries, in plain English, which are sent to us; we will do our best to translate them into SQL and even incorporate them as predefined questions if they prove to be of general interest.

### Examples of advanced queries

Often a researcher’s question cannot be answered using any of the seven predefined queries. Nevertheless, to provide, from the beginning, a way to make any query, knowledgeable users can access a SQL console where they can write any unrestricted query. Since all the contents of the database come from public information, and no personal or sensitive data are stored, this does not represent a legal or privacy problem. Also, since users are allowed to access the database in read-only mode, integrity is not a concern, either. Notice that very few biomedical databases allow this unrestricted access.

Thus, new questions can be asked using advanced queries. For instance, how many types of cancer can be found in the database and how often do they appear? To do this, the input in the advanced query would be:

SELECT patients_affected_by.IdPathologyCD10, Pathology.Name, COUNT(patients_affected_by.IdPathologyCD10) FROM patients_affected_by,Pathology WHERE patients_affected_by.IdPathologyCD10= Pathology.IdPathologyCD10 GROUP BY patients_affected_by.IdPathologyCD10 ORDER BY COUNT(patients_affected_by.IdPathologyCD10) DESC

The output corresponds to the 23 different types of cancer with their ICD10 codes, names and frequencies with which they are reported. The five most frequent types are shown in Table 3.

Another example of an advanced query is: what is the average percentage of cardiotoxicity reported in all the studies which have measured it?

The answer is 14.4 % and to obtain this the following advanced query needs to be introduced:

SELECT SUM(ObservedCardiotoxicity*NumSubjects)/ (SUM(NumSubjects)) FROM Study WHERE ObservedCardiotoxicity IS NOT NULL;

Notice that this result has been calculated weighting the cardiotoxicity percentages reported in each study proportionally to the number of subjects in it with respect to the total number of subjects.

Another interesting question is: which are the top three variations in the database? To answer this, the following advanced query needs to be used:

SELECT IdVariation, COUNT(IdVariation) FROM variation_studied_by GROUP BY IdVariation ORDER BY COUNT(IdVariation) DESC LIMIT 3;

the most frequent being natriuretic peptide B (frequency = 20), troponin T (frequency = 15) and troponin I (frequency = 14).

## Discussion

The area of onco-cardiology is in its early years of study regarding the molecular relationship between cancer and CVDs. This makes possible the collection of all the information in this regard early. The OncoCardioDB designed in this work allows storing all the molecular information obtained from patients in the area of onco-cardiology, incorporating new relevant studies on a regular basis. The global burden of cancer and CVDs continues to increase, and therefore the growing number of patients who survive cancer have an increasing risk of developing CVDs. This is why we think that the OncoCardio database will be an important contribution to identify molecular targets related to both diseases. Concrete applications that can be mentioned are in precision medicine, e.g. when designing new targeted drugs with fewer cardiotoxic effects, or as a repository of possible molecular biomarkers in onco-cardiology. The above comment is based on the fact that the OncoCardio database contains information on all the molecular targets (variations) identified to date associated with cancer that cause a cardiovascular effect, whether due to cardiotoxicity or other reasons.

Another important aspect to be discussed is the usability of the system. It will be really useful only as long as the biomedical community finds it valuable and accessible. Although it is not especially aesthetic, the authors have made an effort to provide simple access and allow only one course of action at any time, so the interface is robust and can be extended or adapted very easily according to the user’s requirements. An important point is the addition of predefined queries on demand; due to the modular organization, this will require the alteration of only a restricted part of the GUI, which is a simple task.

The generation of the SQL sentences to fill the DB in the case of the generic tables (those whose information comes from predefined lists or standards), especially gene-related ones, is relatively slow (it takes ~10 min on a normal modern computer) and its introduction into the database (the call to the generated SQL macro) is even slower (~20 min), but this only has to be done once and is valid forever. On the other hand, the generation of the sentences for the specific tables (namely, the studies and their related values such as observed symptoms and pathologies) and their loading into the database is almost instantaneous, so the addition of further studies to update the information is not a problem.

Apart from this, probably the main drawback for complete user-friendliness is the need to introduce the entities such as genes, pathologies and drugs using their normalized designations exclusively (Entrez code, ATC and ICD10 codes), instead of common or normally accepted names. The use of fully standardized vocabularies is an almost universally acknowledged need since automatic information processing was introduced in medicine. Nevertheless, this is something that is not always accepted by the medical community enthusiastically. It is our opinion that an effort should be carried out by both parties (doctors and information processing experts) to reduce this gap. To that aim, a proposal for future work is provided in the ‘Future work’ section.

Extendibility and reusability are important aspects to be mentioned, too. The GUI is a Java application in which the model interacts with a MariaDB server to make any query and return any result. This makes it possible to use it in any database application with few changes; e.g., more predefined queries can be added without much effort. On the other hand, the controller and the views have been devised for this application and even though they are a good basis for similar ones, a substantial part of their code would have to be changed.

Biological knowledge is completely contained inside the database and to some extent can be reused. Tables containing general entities (genes, drugs and pathologies) can be exported separately and used as such for other medical databases. Reorganization of the database by adding fields, or even new tables, is possible, too, but may require rewriting of one or more predefined queries.

Finally, the authentication and user-management parts are the most reusable components. They are completely independent of the GUI and the database; indeed, they can work with any other program where access is to be offered through the network with just a browser, even if it is not a user interface or a database.

### Future work

The system is functional and awaits the interaction of interested users. Their suggestions and comments will be important to introduce improvements, but some of them are already planned, namely:

- In the case that the user does not know the ICD10 code of a pathology but only its name, a search through the ICD10 database by similar words or expressions will be provided. The similarity between character strings, including metrics, to show possible matches ordered by closeness is a habitual task in natural language processing, and several libraries aimed at this objective are available.
- The interface to look for pathology codes can be organized, too, based on a rational taxonomy of diseases, but this is already provided by the ICD10 web (see https://www.icd10data.com/ICD10CM/Codes) which has been made accessible as part of the GUI of the system.
- A similar strategy can be applied to the ATC codes of drugs. In this case, the rational taxonomy is available at https://www.atccode.com/ and is also available in our system.
- A program option is currently implemented which allows authors of papers on onco-cardiology to inform us about their works so that the content can be incorporated into the database. It will also be possible to provide a form where the authors themselves can fill in the values of each of the relevant fields mentioned in their work. Such information will be curated in its form by the syntactic checks mentioned in the section ‘Knowledge extraction and curation’ and in its content by the database maintainers.

With respect to the system’s organization and the experience acquired during its construction, it is likely that can be used to build similar systems in other biomedical areas. The system is sufficiently modular, and in particular, the organization of the user interface and the orchestration of all the parts under Guacamole are fully reusable.

In a similar way, some of the tables of the database can be used without changes in applications that require these data (drug names/codes or information on genes and variations).

## Supplementary Material

baad029_Supp

## Data Availability

The database can be consulted through a web browser, but for those wishing to reproduce this work or to use the data for other purposes, our software is available under a free GNU licence. This includes an R script to fill in the gene-related tables from data in the Bioconductor package org.Hs.eg.db and Perl scripts to fill in the drug-related tables from the ATC as provided by the WHO Collaborating Centre for Drug Statistics Methodology of Norway (23) and to fill in the pathology-related tables from the ICD10 as provided by the Web’s Free 2022 ICD-10-CM/PCS Medical Coding Reference (24). The distribution of the complete populated database as files loadable by a database system such as MariaDB or MySQL is possible, too, since the data contained in it are publicly available. The R package org.Hs.eg.db is distributed under Artistic License 2.0. With respect to the ATC and ICD10, the authors acknowledge, in their respective pages, the public availability of the provided information. To facilitate adaptation and reuse, we installed, and are currently running, the system inside a virtual machine created with QEMU/Kernel Virtual Machine (integrated inside the Linux kernel) which runs a GNU/Linux operating system. GNU/Linux and MariaDB are distributed under the terms of the GNU General Public License (GPL). Guacamole is available under the Apache License. Concerning the software produced by the authors (GUI in Java, Perl macros of data treatment and Perl daemon), this is distributed by us on demand in source form under the GPL, too. Therefore, there is no legal obstacle to making the full virtual machine available. It can be downloaded from https://johnford.uv.es/OncocardioVM and ported to another domain following the directions on the download page. Obviously, it is also possible to install and run each part of the system in a real machine.
