# Supplementary material for: OncoCardioDB: a public and curated database of molecular information in onco-cardiology/cardio-oncology
Source: Database (Oxford). 2023 May 9;2023:baad029. doi: 10.1093/database/baad029 (PMC10167979; doi:10.1093/database/baad029)
Supplement: baad029_Supp [file baad029_supp.zip › suppl_data/ocsupplementary.pdf]

References of the papers used to populate the OncoCardio database with their accessible URLs and search strategies used

Supplementary material 1 to the paper "OncoCardioDB: A Public and Curated Database of Molecular Information in Onco-cardiology/Cardio-oncology"

A.L. Riffo-Campos, J Domingo, E. Dura
(angela.riffo@ufrontera.cl, Juan.Domingo@uv.es, Esther.Dura@uv.es)

[1] Mahmut Akpek et al. "Protective effects of spironolactone against anthracycline-induced cardiomyopathy". In: *European Journal of Heart Failure* 17.1 (Jan. 2015), pp. 81–89. ISSN: 13889842. DOI: 10.1002/ejhf.196. URL: https://onlinelibrary.wiley.com/doi/10.1002/ejhf.196.

[2] Raza M. Alvi et al. "Cardiovascular Events Among Adults Treated With Chimeric Antigen Receptor T-Cells (CAR-T)". In: *Journal of the American College of Cardiology* 74.25 (Dec. 2019), pp. 3099–3108. ISSN: 07351097. DOI: 10.1016/j.jacc.2019.10.038. URL: https://reader.elsevier.com/reader/sd/pii/S0735109719382658.

[3] Henrike Arfsten et al. "GDF-15 in solid vs non-solid treatment-naïve malignancies". In: *European Journal of Clinical Investigation* 49.11 (Nov. 2019), p. 13168. ISSN: 0014-2972. DOI: 10.1111/eci.13168. URL: https://doi.org/10.1111/eci.13168wileyonlinelibrary.com/journal/eci%20https://onlinelibrary.wiley.com/doi/10.1111/eci.13168.

[4] Aarti Asnani et al. "Changes in Citric Acid Cycle and Nucleoside Metabolism Are Associated with Anthracycline Cardiotoxicity in Patients with Breast Cancer". In: *Journal of Cardiovascular Translational Research* 13.3 (June 2020), pp. 349–356. ISSN: 1937-5387. DOI: 10.1007/s12265-019-09897-y. URL: https://doi.org/10.1007/s12265-019-09897-y%20http://link.springer.com/10.1007/s12265-019-09897-y.

[5] Hanna Aula et al. "Decreases in TGF-β1 and PDGF levels are associated with echocardiographic changes during adjuvant radiotherapy for breast cancer". In: *Radiation Oncology* 13.1 (Dec. 2018), p. 201. ISSN: 1748-717X. DOI: 10.1186/s13014-018-1150-7. URL: https://ro-journal.biomedcentral.com/articles/10.1186/s13014-018-1150-7.

[6] Hanna Aula et al. "ST2 levels increased and were associated with changes in left ventricular systolic function during a three-year follow-up after adjuvant radiotherapy for breast cancer". In: *The Breast* 49 (Feb. 2020), pp. 183–186. ISSN: 09609776. DOI: 10.1016/j.breast.2019.12.001. URL: https://reader.elsevier.com/reader/sd/pii/S0960977619311063.

[7] Hanna Aula et al. "Transforming growth factor beta 1 levels predict echocardiographic changes at three years after adjuvant radiotherapy for breast cancer". In: *Radiation Oncology* 14.1 (Dec. 2019), p. 155. ISSN: 1748-717X. DOI: 10.1186/s13014-019-1366-1. URL: https://ro-journal.biomedcentral.com/articles/10.1186/s13014-019-1366-1.

[8] Lynn A. Beer et al. "Baseline Immunoglobulin E Levels as a Marker of Doxorubicin- and Trastuzumab-Associated Cardiac Dysfunction". In: *Circulation Research* 119.10 (Oct. 2016), pp. 1135–1144. ISSN: 0009-7330. DOI: 10.1161/CIRCRESAHA.116.309004. URL: https://www.ahajournals.org/doi/abs/10.1161/CIRCRESAHA.116.309004%20https://www.ahajournals.org/doi/10.1161/CIRCRESAHA.116.309004.

[9] Annelies H. Boekhout et al. "Angiotensin II–Receptor Inhibition With Candesartan to Prevent Trastuzumab-Related Cardiotoxic Effects in Patients With Early Breast Cancer". In: *JAMA Oncology* 2.8 (Aug. 2016), p. 1030. ISSN: 2374-2437. DOI: 10.1001/jamaoncol.2016.1726. URL: http://oncology.jamanetwork.com/article.aspx?doi=10.1001/jamaoncol.2016.1726.

[10] W. van Boxtel et al. "New biomarkers for early detection of cardiotoxicity after treatment with docetaxel, doxorubicin and cyclophosphamide". In: *Biomarkers* 20.2 (Feb. 2015), pp. 143–148. ISSN: 1354-750X. DOI: 10.3109/1354750X.2015.1040839. URL: https://www.tandfonline.com/doi/abs/10.3109/1354750X.2015.1040839%20https://www.tandfonline.com/doi/full/10.3109/1354750X.2015.1040839.

[11] Vito Calabrese et al. "Early Diastolic Dysfunction after Cancer Chemotherapy: Primary Endpoint Results of a Multicenter Cardio-Oncology Study". In: *Chemotherapy* 63.2 (Feb. 2018), pp. 55–63. ISSN: 0009-3157. DOI: 10.1159/000486761. URL: https://pubmed.ncbi.nlm.nih.gov/29428939/%20https://www.karger.com/Article/FullText/486761.

[12] Justin M. Canada et al. "Increased C-reactive protein is associated with the severity of thoracic radiotherapy-induced cardiomyopathy". In: *Cardio-Oncology* 6.1 (Dec. 2020), p. 2. ISSN: 2057-3804. DOI: 10.1186/s40959-020-0058-1. URL: https://cardiooncologyjournal.biomedcentral.com/articles/10.1186/s40959-020-0058-1.

[13] Daniela Cardinale et al. "Prevention of High-Dose Chemotherapy–Induced Cardiotoxicity in High-Risk Patients by Angiotensin-Converting Enzyme Inhibition". In: *Circulation* 114.23 (Dec. 2006), pp. 2474–2481. ISSN: 0009-7322. DOI: 10.1161/CIRCULATIONAHA.106.635144. URL: https://www.ahajournals.org/doi/abs/10.1161/circulationaha.106.635144%20https://www.ahajournals.org/doi/10.1161/CIRCULATIONAHA.106.635144.

[14] Daniela Cardinale et al. "Prognostic Value of Troponin I in Cardiac Risk Stratification of Cancer Patients Undergoing High-Dose Chemotherapy". In: *Circulation* 109.22 (June 2004), pp. 2749–2754. ISSN: 0009-7322. DOI: 10.1161/01.CIR.0000130926.51766.CC. URL: http://www.circulationaha.org%20https://www.ahajournals.org/doi/10.1161/01.CIR.0000130926.51766.CC.

[15] Almudena Cascales et al. "Association of Anthracycline-Related Cardiac Histological Lesions With NADPH Oxidase Functional Polymorphisms". In: *The Oncologist* 18.4 (Apr. 2013), pp. 446–453. ISSN: 1083-7159. DOI: 10.1634/theoncologist.2012-0239. URL: https://pericles-gcp.literatumonline.com/doi/10.1634/theoncologist.2012-0239.

[16] Jianqiong Chen et al. "Early detection of cardiotoxicity by 3D speckle tracking imaging of area strain in breast cancer patients receiving chemotherapy". In: *Echocardiography* 36.9 (Sept. 2019), pp. 1682–1688. ISSN: 0742-2822. DOI: 10.1111/echo.14467. URL: https://onlinelibrary.wiley.com/doi/full/10.1111/echo.14467%20https://onlinelibrary.wiley.com/doi/abs/10.1111/echo.14467%20https://onlinelibrary.wiley.com/doi/10.1111/echo.14467.

[17] Kalyan R. Chitturi et al. "Immune Checkpoint Inhibitor-Related Adverse Cardiovascular Events in Patients With Lung Cancer". In: *JACC: CardioOncology* 1.2 (Dec. 2019), pp. 182–192. ISSN: 26660873. DOI: 10.1016/j.jaccao.2019.11.013. URL: https://reader.elsevier.com/reader/sd/pii/S2666087319301000.

[18] Timucin Cil et al. "Use of N-Terminal Pro-Brain Natriuretic Peptide to Assess Left Ventricular Function after Adjuvant Doxorubicin Therapy in Early Breast Cancer Patients". In: *Clinical Drug Investigation* 29.2 (2009), pp. 131–137. ISSN: 1173-2563. DOI: 10.2165/0044011-200929020-00007. URL: http://link.springer.com/10.2165/0044011-200929020-00007.

[19] Maria Patrizia D'Errico et al. "Kinetics of B-type natriuretic peptide plasma levels in patients with left-sided breast cancer treated with radiation therapy: Results after one-year follow-up". In: *International Journal of Radiation Biology* 91.10 (Oct. 2015), pp. 804–809. ISSN: 0955-3002. DOI: 10.3109/09553002.2015.1027421. URL: http://www.tandfonline.com/doi/full/10.3109/09553002.2015.1027421.

[20] Dong Dai et al. "Correlation of fluorine 18-labeled sodium fluoride uptake and arterial calcification on whole-body PET/CT in cancer patients". In: *Nuclear Medicine Communications* 40.6 (June 2019), pp. 604–610. ISSN: 0143-3636. DOI: 10.1097/MNM.0000000000000986. URL: https://pubmed.ncbi.nlm.nih.gov/30688757/%20https://journals.lww.com/00006231-201906000-00008.

[21] Francesca De Iuliis et al. "Serum biomarkers evaluation to predict chemotherapy-induced cardiotoxicity in breast cancer patients". In: *Tumor Biology* 37.3 (Mar. 2016), pp. 3379–3387. ISSN: 1010-4283. DOI: 10.1007/s13277-015-4183-7. URL: http://link.springer.com/10.1007/s13277-015-4183-7.

[22] Aurelia H. M. de Vries Schultink et al. "Pharmacodynamic modeling of cardiac biomarkers in breast cancer patients treated with anthracycline and trastuzumab regimens". In: *Journal of Pharmacokinetics and Pharmacodynamics* 45.3 (June 2018), pp. 431–442. ISSN: 1567-567X. DOI: 10.1007/s10928-018-9579-8. URL: https://doi.org/10.1007/s10928-018-9579-8%20http://link.springer.com/10.1007/s10928-018-9579-8.

[23] Biniyam G. Demissei et al. “Changes in Cardiovascular Biomarkers With Breast Cancer Therapy and Associations With Cardiac Dysfunction”. In: *Journal of the American Heart Association* 9.2 (Jan. 2020). ISSN: 2047-9980. DOI: 10.1161/JAHA.119.014708. URL: https://www.ahajournals.org/doi/abs/10.1161/JAHA.119.014708%20https://www.ahajournals.org/doi/10.1161/JAHA.119.014708.

[24] Jia-Yin Di, Zong-Xin Zhang, and Shao-Jun Xin. “Glycogen Phosphorylase Isoenzyme Bb, Myoglobin and BNP in ANT-Induced Cardiotoxicity”. In: *Open Life Sciences* 13.1 (Dec. 2018), pp. 561–568. ISSN: 2391-5412. DOI: 10.1515/biol-2018-0067. URL: /pmc/articles/PMC7874740/%20/pmc/articles/PMC7874740/?report=abstract%20https://www.ncbi.nlm.nih.gov/pmc/articles/PMC7874740/%20https://www.degruyter.com/document/doi/10.1515/biol-2018-0067/html.

[25] Daniel A. Duprez et al. “Comparison of the Predictive Value of GlycA and Other Biomarkers of Inflammation for Total Death, Incident Cardiovascular Events, Noncardiovascular and Noncancer Inflammatory-Related Events, and Total Cancer Events”. In: *Clinical Chemistry* 62.7 (July 2016), pp. 1020–1031. ISSN: 0009-9147. DOI: 10.1373/clinchem.2016.255828. URL: https://academic.oup.com/clinchem/article/62/7/1020/5611895.

[26] Refik Erdim et al. “Cardiac troponin T for early detection of cardiotoxicity in breast cancer patients treated with epirubicin”. In: *Open Medicine* 4.3 (Sept. 2009), pp. 327–330. ISSN: 2391-5463. DOI: 10.2478/s11536-008-0093-z. URL: https://www.degruyter.com/document/doi/10.2478/s11536-008-0093-z/html.

[27] Hamidreza Fasehee et al. “Cancer biomarkers in atherosclerotic plaque: Evidenced from structural and proteomic analyses”. In: *Biochemical and Biophysical Research Communications* 509.3 (Feb. 2019), pp. 687–693. ISSN: 0006291X. DOI: 10.1016/j.bbrc.2018.12.160. URL: https://linkinghub.elsevier.com/retrieve/pii/S0006291X18328419.

[28] Brian S. Finkelman et al. “Arginine-Nitric Oxide Metabolites and Cardiac Dysfunction in Patients With Breast Cancer”. In: *Journal of the American College of Cardiology* 70.2 (July 2017), pp. 152–162. ISSN: 07351097. DOI: 10.1016/j.jacc.2017.05.019. URL: https://reader.elsevier.com/reader/sd/pii/S0735109717373643.

[29] Roberta Florido et al. “Cancer Survivorship and Subclinical Myocardial Damage”. In: *American Journal of Epidemiology* 188.12 (Dec. 2019), pp. 2188–2195. ISSN: 0002-9262. DOI: 10.1093/aje/kwz088. URL: https://academic.oup.com/aje/article/188/12/2188/5423751.

[30] Pierre Frères et al. “Variations of circulating cardiac biomarkers during and after anthracycline-containing chemotherapy in breast cancer patients”. In: *BMC Cancer* 18.1 (Dec. 2018), p. 102. ISSN: 1471-2407. DOI: 10.1186/s12885-018-4015-4. URL: https://bmccancer.biomedcentral.com/articles/10.1186/s12885-018-4015-4.

[31] Michael A. Fridrik et al. “Cardiotoxicity with rituximab, cyclophosphamide, non-pegylated liposomal doxorubicin, vincristine and prednisolone compared to rituximab, cyclophosphamide, doxorubicin, vincristine, and prednisolone in frontline treatment of patients with diffuse large B-”. In: *European Journal of Cancer* 58 (May 2016), pp. 112–121. ISSN: 09598049. DOI: 10.1016/j.ejca.2016.02.004. URL: http://www.ejcancer.com/article/S0959804916000885/fulltext%20http://www.ejcancer.com/article/S0959804916000885/abstract%20https://www.ejcancer.com/article/S0959-8049(16)00088-5/abstract%20https://linkinghub.elsevier.com/retrieve/pii/S0959804916000885.

[32] Ornella Garrone et al. “Prediction of Anthracycline Cardiotoxicity after Chemotherapy by Biomarkers Kinetic Analysis”. In: *Cardiovascular Toxicology* 12.2 (June 2012), pp. 135–142. ISSN: 1530-7905. DOI: 10.1007/s12012-011-9149-4. URL: http://link.springer.com/10.1007/s12012-011-9149-4.

[33] Sandra Geiger et al. “Anthracycline-Induced Cardiotoxicity: Cardiac Monitoring by Continuous Wave-Doppler Ultrasound Cardiac Output Monitoring and Correlation to Echocardiography”. In: *Onkologie* 35.5 (May 2012), pp. 241–246. ISSN: 1423-0240. DOI: 10.1159/000338335. URL: https://www.karger.com/Article/FullText/338335%20https://www.karger.com/Article/Abstract/338335.

[34] Carrie A Geisberg et al. “Circulating Neuregulin During the Transition From Stage A to Stage B/C Heart Failure in a Breast Cancer Cohort”. In: *Journal of Cardiac Failure* 19.1 (Jan. 2013), pp. 10–15. ISSN: 10719164. DOI: 10.1016/j.cardfail.2012.11.006. URL: https://linkinghub.elsevier.com/retrieve/pii/S1071916412013619.

[35] Celia Gómez Peña et al. "Influence of the HER2 Ile655Val polymorphism on trastuzumab-induced cardiotoxicity in HER2-positive breast cancer patients". In: *Pharmacogenetics and Genomics* 25.8 (Aug. 2015), pp. 388–393. ISSN: 1744-6872. DOI: 10.1097/FPC.0000000000000149. URL: https://pubmed.ncbi.nlm.nih.gov/26049584/%20https://journals.lww.com/01213011-201508000-00003.

[36] E. Wilson Grandin et al. "Patterns of Cardiac Toxicity Associated With Irreversible Proteasome Inhibition in the Treatment of Multiple Myeloma". In: *Journal of Cardiac Failure* 21.2 (Feb. 2015), pp. 138–144. ISSN: 10719164. DOI: 10.1016/j.cardfail.2014.11.008. URL: http://www.onlinejcf.com/article/S1071916414013074/fulltext%20http://www.onlinejcf.com/article/S1071916414013074/abstract%20https://www.onlinejcf.com/article/S1071-9164(14)01307-4/abstract%20https://linkinghub.elsevier.com/retrieve/pii/S1071916414013074.

[37] Geeta Gulati et al. "Neurohormonal Blockade and Circulating Cardiovascular Biomarkers During Anthracycline Therapy in Breast Cancer Patients: Results From the PRADA (Prevention of Cardiac Dysfunction During Adjuvant Breast Cancer Therapy) Study". In: *Journal of the American Heart Association* 6.11 (Nov. 2017). ISSN: 2047-9980. DOI: 10.1161/JAHA.117.006513. URL: https://www.ahajournals.org/doi/abs/10.1161/JAHA.117.006513%20https://www.ahajournals.org/doi/10.1161/JAHA.117.006513.

[38] K. Sundby Hall et al. "Effects of dose-intensive chemotherapy and radiotherapy on serum n-terminal proatrial natriuretic peptide in high-risk breast cancer patients". In: *Breast Cancer Research and Treatment* 67.3 (June 2001), pp. 235–244. ISSN: 0167-6806. DOI: 10.1023/A:1017946315032. URL: http://link.springer.com/10.1023/A:1017946315032.

[39] Martin Halle, Tinna Christersdottir, and Magnus Bfäck. "Chronic adventitial inflammation, vasa vasorum expansion, and 5-lipoxygenase up-regulation in irradiated arteries from cancer survivors". In: *The FASEB Journal* 30.11 (Nov. 2016), pp. 3845–3852. ISSN: 0892-6638. DOI: 10.1096/fj.201600620R. URL: https://onlinelibrary.wiley.com/doi/abs/10.1096/fj.201600620R.

[40] Jasmin D. Haslbauer et al. "CMR imaging biosignature of cardiac involvement due to cancer-related treatment by T1 and T2 mapping". In: *International Journal of Cardiology* 275 (Jan. 2019), pp. 179–186. ISSN: 01675273. DOI: 10.1016/j.ijcard.2018.10.023. URL: http://www.internationaljournalofcardiology.com/article/S0167527318349921/fulltext%20http://www.internationaljournalofcardiology.com/article/S0167527318349921/abstract%20https://www.internationaljournalofcardiology.com/article/S0167-5273(18)34992-1/abstract%20h.

[41] Peter G Hawkins et al. "Circulating microRNAs as biomarkers of radiation-induced cardiac toxicity in non-small-cell lung cancer". In: *Journal of Cancer Research and Clinical Oncology* 145.6 (June 2019), pp. 1635–1643. ISSN: 0171-5216. DOI: 10.1007/s00432-019-02903-5. URL: https://doi.org/10.1007/s00432-019-02903-5%20http://link.springer.com/10.1007/s00432-019-02903-5.

[42] Claes Held et al. "Inflammatory Biomarkers Interleukin-6 and C-Reactive Protein and Outcomes in Stable Coronary Heart Disease: Experiences From the STABILITY (Stabilization of Atherosclerotic Plaque by Initiation of Darapladib Therapy) Trial". In: *Journal of the American Heart Association* 6.10 (Oct. 2017). ISSN: 2047-9980. DOI: 10.1161/JAHA.116.005077. URL: https://www.ahajournals.org/doi/abs/10.1161/JAHA.116.005077%20https://www.ahajournals.org/doi/10.1161/JAHA.116.005077.

[43] Joerg Herrmann, Sandra M. Herrmann, and Tufia C. Haddad. "New-Onset Heart Failure in Association With Severe Hypertension During Trastuzumab Therapy". In: *Mayo Clinic Proceedings* 89.12 (Dec. 2014), pp. 1734–1739. ISSN: 00256196. DOI: 10.1016/j.mayocp.2014.08.011. URL: https://pubmed.ncbi.nlm.nih.gov/25441402/%20https://linkinghub.elsevier.com/retrieve/pii/S0025619614007459.

[44] Daniel L. Hertz et al. "Evidence for association of SNPs in ABCB1 and CBR3 , but not RAC2, NCF4, SLC28A3 or TOP2B , with chronic cardiotoxicity in a cohort of breast cancer patients treated with anthracyclines". In: *Pharmacogenomics* 17.3 (Feb. 2016), pp. 231–240. ISSN: 1462-2416. DOI: 10.2217/pgs.15.162. URL: https://pubmed.ncbi.nlm.nih.gov/26799497/%20https://www.futuremedicine.com/doi/10.2217/pgs.15.162.

[45] Melissa Jones et al. "High-sensitive cardiac troponin-I facilitates timely detection of subclinical anthracycline-mediated cardiac injury". In: *Annals of Clinical Biochemistry: International Journal of Laboratory Medicine* 54.1 (Jan. 2017), pp. 149–157. ISSN: 0004-5632. DOI: 10.1177/0004563216650464. URL: http://journals.sagepub.com/doi/10.1177/0004563216650464.

[46] Hana Kalábová et al. "Intima-media thickness, myocardial perfusion and laboratory risk factors of atherosclerosis in patients with breast cancer treated with anthracycline-based chemotherapy". In: *Medical Oncology* 28.4 (Dec. 2011), pp. 1281–1287. ISSN: 1357-0560. DOI: 10.1007/s12032-010-9593-1. URL: http://link.springer.com/10.1007/s12032-010-9593-1.

[47] Kenichi Katsurada et al. "High-sensitivity troponin T as a marker to predict cardiotoxicity in breast cancer patients with adjuvant trastuzumab therapy". In: *SpringerPlus* 3.1 (Dec. 2014), p. 620. ISSN: 2193-1801. DOI: 10.1186/2193-1801-3-620. URL: http://www.springerplus.com/content/3/1/620%20https://springerplus.springeropen.com/articles/10.1186/2193-1801-3-620.

[48] Elham Kazemian et al. "Vitamin D receptor gene polymorphisms affecting changes in visceral fat, waist circumference and lipid profile in breast cancer survivors supplemented with vitamin D3". In: *Lipids in Health and Disease* 18.1 (Dec. 2019), p. 161. ISSN: 1476-511X. DOI: 10.1186/s12944-019-1100-x. URL: https://lipidworld.biomedcentral.com/articles/10.1186/s12944-019-1100-x.

[49] Hiromitsu Kitayama et al. "High-sensitive troponin T assay can predict anthracycline- and trastuzumab-induced cardiotoxicity in breast cancer patients". In: *Breast Cancer* 24.6 (Nov. 2017), pp. 774–782. ISSN: 1340-6868. DOI: 10.1007/s12282-017-0778-8. URL: https://link.springer.com/article/10.1007/s12282-017-0778-8%20http://link.springer.com/10.1007/s12282-017-0778-8%20http://link.springer.com/10.1007/s13277-014-1679-5.

[50] Annop Kittiwarawut et al. "Serum NT-proBNP in the early detection of doxorubicin-induced cardiac dysfunction". In: *Asia-Pacific Journal of Clinical Oncology* 9.2 (June 2013), pp. 155–161. ISSN: 17437555. DOI: 10.1111/j.1743-7563.2012.01588.x. URL: https://pubmed.ncbi.nlm.nih.gov/22897825/%20https://onlinelibrary.wiley.com/doi/10.1111/j.1743-7563.2012.01588.x.

[51] Alexandros Kouloubinis et al. "The role of TNF-α, Fas/Fas ligand system and NT-proBNP in the early detection of asymptomatic left ventricular dysfunction in cancer patients treated with anthracyclines". In: *IJC Heart & Vasculature* 6.8 (Mar. 2015), pp. 85–90. ISSN: 23529067. DOI: 10.1016/j.ijcha.2015.01.002. URL: https://reader.elsevier.com/reader/sd/pii/S2352906715000032.

[52] Jock Chichaco Kuruc et al. "Development of anthracycline-induced dilated cardiomyopathy due to mutation on LMNA gene in a breast cancer patient: a case report". In: *BMC Cardiovascular Disorders* 19.1 (Dec. 2019), p. 169. ISSN: 1471-2261. DOI: 10.1186/s12872-019-1155-7. URL: https://bmccardiovascdisord.biomedcentral.com/articles/10.1186/s12872-019-1155-7.

[53] Bonnie Ky et al. "Early Increases in Multiple Biomarkers Predict Subsequent Cardiotoxicity in Patients With Breast Cancer Treated With Doxorubicin, Taxanes, and Trastuzumab". In: *Journal of the American College of Cardiology* 63.8 (Mar. 2014), pp. 809–816. ISSN: 07351097. DOI: 10.1016/j.jacc.2013.10.061. URL: https://reader.elsevier.com/reader/sd/pii/S0735109713061573.

[54] Hai Li et al. "Correlation of UGT2B7 Polymorphism with Cardiotoxicity in Breast Cancer Patients Undergoing Epirubicin/Cyclophosphamide-Docetaxel Adjuvant Chemotherapy". In: *Yonsei Medical Journal* 60.1 (Jan. 2019), p. 30. ISSN: 0513-5796. DOI: 10.3349/ymj.2019.60.1.30. URL: https://doi.org/10.3349/ymj.2019.60.1.30%20https://eymj.org/DOIx.php?id=10.3349/ymj.2019.60.1.30.

[55] M. Linschoten et al. "Truncating Titin (TTN) Variants in Chemotherapy-Induced Cardiomyopathy". In: *Journal of Cardiac Failure* 23.6 (June 2017), pp. 476–479. ISSN: 10719164. DOI: 10.1016/j.cardfail.2017.03.003. URL: https://reader.elsevier.com/reader/sd/pii/S1071916417300635.

[56] Joanna M. Lubieniecka et al. "A discovery study of daunorubicin induced cardiotoxicity in a sample of acute myeloid leukemia patients prioritizes P450 oxidoreductase polymorphisms as a potential risk factor". In: *Frontiers in Genetics* 4.NOV (2013). ISSN: 1664-8021. DOI: 10.3389/fgene.2013.00231. URL: http://journal.frontiersin.org/article/10.3389/fgene.2013.00231/abstract.

[57] Abhidha Malik et al. "Are Biomarkers Predictive of Anthracycline-Induced Cardiac Dysfunction?" In: *Asian Pacific Journal of Cancer Prevention* 17.4 (June 2016), pp. 2301–2305. ISSN: 1513-7368. DOI: 10.7314/APJCP.2016.17.4.2301. URL: http://koreascience.or.kr/journal/view.jsp?kj=POCPA9&py=2016&vnc=v17n4&sp=2301.

[58] V Michalaki et al. "Evaluation of serum lipids and high-density lipoprotein subfractions (HDL2, HDL3) in postmenopausal patients with breast cancer". In: *Molecular and Cellular Biochemistry* 268.1-2 (Jan. 2005), pp. 19–24. ISSN: 0300-8177. DOI: 10.1007/s11010-005-2993-4. URL: http://link.springer.com/10.1007/s11010-005-2993-4.

[59] Seiichi Mokuyasu et al. “High-sensitivity cardiac troponin I detection for 2 types of drug-induced cardiotoxicity in patients with breast cancer”. In: *Breast Cancer* 22.6 (Nov. 2015), pp. 563–569. ISSN: 1340-6868. DOI: 10.1007/s12282-014-0520-8. URL: http://link.springer.com/10.1007/s12282-014-0520-8.

[60] Cristian Mornoş and Lucian Petrescu. “Early detection of anthracycline-mediated cardiotoxicity: the value of considering both global longitudinal left ventricular strain and twist”. In: *Canadian Journal of Physiology and Pharmacology* 91.8 (Aug. 2013), pp. 601–607. ISSN: 0008-4212. DOI: 10.1139/cjpp-2012-0398. URL: https://cdnsciencepub.com/doi/abs/10.1139/cjpp-2012-0398%20http://www.nrcresearchpress.com/doi/10.1139/cjpp-2012-0398.

[61] L Mortara et al. “Efficacy of sorafenib and impact on cardiac function in patients with thyroid cancer: a retrospective analysis”. In: *Journal of Endocrinological Investigation* 37.11 (Nov. 2014), pp. 1099–1108. ISSN: 1720-8386. DOI: 10.1007/s40618-014-0177-3. URL: http://link.springer.com/10.1007/s13277-015-4183-7%20http://link.springer.com/10.1007/s40618-014-0177-3.

[62] G Murtagh et al. “Late cardiac effects of chemotherapy in breast cancer survivors treated with adjuvant doxorubicin: 10-year follow-up”. In: *Breast Cancer Research and Treatment* 156.3 (Apr. 2016), pp. 501–506. ISSN: 0167-6806. DOI: 10.1007/s10549-016-3781-4. URL: http://www.r-pro%20http://link.springer.com/10.1007/s10549-016-3781-4.

[63] Mari Hara Nakano et al. “A Genome-Wide Association Study Identifies Five Novel Genetic Markers for Trastuzumab-Induced Cardiotoxicity in Japanese Population”. In: *Biological and Pharmaceutical Bulletin* 42.12 (Dec. 2019), pp. 2045–2053. ISSN: 0918-6158. DOI: 10.1248/bpb.b19-00527. URL: http://locuszoom.sph.umich.edu/%20https://www.jstage.jst.go.jp/article/bpb/42/12/42_b19-00527/_article.

[64] Matthew Ng, David Cunningham, and Andrew R. Norman. “The frequency and pattern of cardiotoxicity observed with capecitabine used in conjunction with oxaliplatin in patients treated for advanced colorectal cancer (CRC)”. In: *European Journal of Cancer* 41.11 (July 2005), pp. 1542–1546. ISSN: 09598049. DOI: 10.1016/j.ejca.2005.03.027. URL: http://www.ejcancer.com/article/S0959804905003461/fulltext%20http://www.ejcancer.com/article/S0959804905003461/abstract%20https://www.ejcancer.com/article/S0959-8049(05)00346-1/abstract%20https://linkinghub.elsevier.com/retrieve/pii/S0959804905003461.

[65] J. Nuver et al. “Microalbuminuria, decreased fibrinolysis, and inflammation as early signs of atherosclerosis in long-term survivors of disseminated testicular cancer”. In: *European Journal of Cancer* 40.5 (Mar. 2004), pp. 701–706. ISSN: 09598049. DOI: 10.1016/j.ejca.2003.12.012. URL: http://www.ejcancer.com/article/S0959804904000036/fulltext%20http://www.ejcancer.com/article/S0959804904000036/abstract%20https://www.ejcancer.com/article/S0959-8049(04)00003-6/abstract%20https://linkinghub.elsevier.com/retrieve/pii/S0959804904000036.

[66] Takuya Oikawa et al. “Increased risk of cancer death in patients with chronic heart failure with a special reference to inflammation-A report from the CHART-2 Study”. In: *International Journal of Cardiology* 290 (Sept. 2019), pp. 106–112. ISSN: 01675273. DOI: 10.1016/j.ijcard.2019.04.078. URL: http://www.internationaljournalofcardiology.com/article/S0167527318363228/fulltext%20http://www.internationaljournalofcardiology.com/article/S0167527318363228/abstract%20https://www.internationaljournalofcardiology.com/article/S0167-5273(18)36322-8/abstract%20h.

[67] Jacopo Olivieri et al. “Modern Management of Anthracycline-Induced Cardiotoxicity in Lymphoma Patients: Low Occurrence of Cardiotoxicity with Comprehensive Assessment and Tailored Substitution by Nonpegylated Liposomal Doxorubicin”. In: *The Oncologist* 22.4 (Apr. 2017), pp. 422–431. ISSN: 1083-7159. DOI: 10.1634/theoncologist.2016-0289. URL: https://onlinelibrary.wiley.com/doi/abs/10.1634/theoncologist.2016-0289.

[68] Adedayo A Onitilo et al. “High-sensitivity C-reactive protein (hs-CRP) as a biomarker for trastuzumab-induced cardiotoxicity in HER2-positive early-stage breast cancer: a pilot study”. In: *Breast Cancer Research and Treatment* 134.1 (July 2012), pp. 291–298. ISSN: 0167-6806. DOI: 10.1007/s10549-012-2039-z. URL: http://link.springer.com/10.1007/s10549-012-2039-z.

[69] Alessandro Pastore et al. “Cardiotoxicity After Anthracycline Treatment in Survivors of Adult Cancers: Monitoring by USCOM, Echocardiography and Serum Biomarkers.” In: *World journal of oncology* 4.1 (Feb. 2013), pp. 18–25. ISSN: 1920-454X. DOI: 10.4021/wjon635w. URL: http://www.wjon.org/index.php/wjon/article/view/635%20http://www.ncbi.nlm.nih.gov/pubmed/29147326%20http://www.pubmedcentral.nih.gov/articlerender.fcgi?artid=PMC5649915.

[70] J. Peng et al. "Identification of differential gene expression related to epirubicin-induced cardiomyopathy in breast cancer patients". In: *Human & Experimental Toxicology* 39.4 (Apr. 2020), pp. 393–401. ISSN: 0960-3271. DOI: 10.1177/0960327119893415. URL: https://pubmed.ncbi.nlm.nih.gov/31823667/%20http://journals.sagepub.com/doi/10.1177/0960327119893415.

[71] Patrick J Perik et al. "Serum HER2 levels are increased in patients with chronic heart failure". In: *European Journal of Heart Failure* 9.2 (Feb. 2007), pp. 173–177. ISSN: 13889842. DOI: 10.1016/j.ejheart.2006.05.010. URL: http://doi.wiley.com/10.1016/j.ejheart.2006.05.010.

[72] Andrew Poklepovic et al. "Randomized study of doxorubicin-based chemotherapy regimens, with and without sildenafil, with analysis of intermediate cardiac markers". In: *Cardio-Oncology* 4.1 (Dec. 2018), p. 7. ISSN: 2057-3804. DOI: 10.1186/s40959-018-0033-2. URL: https://cardiooncologyjournal.biomedcentral.com/articles/10.1186/s40959-018-0033-2.

[73] A. L. Pop-Moldovan et al. "Customized laboratory TLR4 and TLR2 detection method from peripheral human blood for early detection of doxorubicin-induced cardiotoxicity". In: *Cancer Gene Therapy* 24.5 (May 2017), pp. 203–207. ISSN: 0929-1903. DOI: 10.1038/cgt.2017.4. URL: https://www.nature.com/articles/cgt20174%20http://www.nature.com/articles/cgt20174.

[74] Mary Putt et al. "Longitudinal Changes in Multiple Biomarkers Are Associated with Cardiotoxicity in Breast Cancer Patients Treated with Doxorubicin, Taxanes, and Trastuzumab". In: *Clinical Chemistry* 61.9 (Sept. 2015), pp. 1164–1172. ISSN: 0009-9147. DOI: 10.1373/clinchem.2015.241232. URL: https://academic.oup.com/clinchem/article/61/9/1164/5611513.

[75] Xiantao Qin et al. "Correlation of circulating pro-angiogenic miRNAs with cardiotoxicity induced by epirubicin/cyclophosphamide followed by docetaxel in patients with breast cancer". In: *Cancer Biomarkers* 23.4 (Dec. 2018), pp. 473–484. ISSN: 18758592. DOI: 10.3233/CBM-181301. URL: https://pubmed.ncbi.nlm.nih.gov/30452398/%20https://www.medra.org/servlet/aliasResolver?alias=iospress&doi=10.3233/CBM-181301.

[76] Paul M Ridker et al. "Effect of interleukin-1β inhibition with canakinumab on incident lung cancer in patients with atherosclerosis: exploratory results from a randomised, double-blind, placebo-controlled trial". In: *The Lancet* 390.10105 (Oct. 2017), pp. 1833–1842. ISSN: 01406736. DOI: 10.1016/S0140-6736(17)32247-X. URL: https://pubmed.ncbi.nlm.nih.gov/28855077/%20https://linkinghub.elsevier.com/retrieve/pii/S014067361732247X.

[77] Vagner Oliveira-Carvalho Rigaud et al. "Circulating miR-1 as a potential biomarker of doxorubicin-induced cardiotoxicity in breast cancer patients". In: *Oncotarget* 8.4 (Jan. 2017), pp. 6994–7002. ISSN: 1949-2553. DOI: 10.18632/oncotarget.14355. URL: https://www.oncotarget.com/article/14355/text/%20https://www.oncotarget.com/article/14355/%20https://www.oncotarget.com/lookup/doi/10.18632/oncotarget.14355.

[78] Lise Roca et al. "Correlation of HER2, FCGR2A, and FCGR3A gene polymorphisms with trastuzumab related cardiac toxicity and efficacy in a subgroup of patients from UNICANCER-PACS04 trial". In: *Breast Cancer Research and Treatment* 139.3 (June 2013), pp. 789–800. ISSN: 0167-6806. DOI: 10.1007/s10549-013-2587-x. URL: http://link.springer.com/10.1007/s10549-013-2587-x.

[79] Luis E. Rohde et al. "Superoxide Dismutase Activity in Adriamycin-Induced Cardiotoxicity in Humans: A Potential Novel Tool for Risk Stratification". In: *Journal of Cardiac Failure* 11.3 (Apr. 2005), pp. 220–226. ISSN: 10719164. DOI: 10.1016/j.cardfail.2004.08.161. URL: http://www.onlinejcf.com/article/S1071916404008498/fulltext%20http://www.onlinejcf.com/article/S1071916404008498/abstract%20https://www.onlinejcf.com/article/S1071-9164(04)00849-8/abstract%20https://linkinghub.elsevier.com/retrieve/pii/S1071916404008498.

[80] D Rossi et al. "Analysis of the host pharmacogenetic background for prediction of outcome and toxicity in diffuse large B-cell lymphoma treated with R-CHOP21". In: *Leukemia* 23.6 (June 2009), pp. 1118–1126. ISSN: 0887-6924. DOI: 10.1038/leu.2008.398. URL: http://www.nature.com/articles/leu2008398.

[81] Sara Ruiz-Pinto et al. "Exome array analysis identifies ETFB as a novel susceptibility gene for anthracycline-induced cardiotoxicity in cancer patients". In: *Breast Cancer Research and Treatment* 167.1 (Jan. 2018), pp. 249–256. ISSN: 0167-6806. DOI: 10.1007/s10549-017-4497-9. URL: https://doi.org/10.1007/s10549-017-4497-9%20http://link.springer.com/10.1007/s10549-017-4497-9.

[82] Bilge Volkan Salanci et al. “The relationship between changes in functional cardiac parameters following anthracycline therapy and carbonyl reductase 3 and glutathione S transferase Pi polymorphisms”. In: *Journal of Chemotherapy* 24.5 (Oct. 2012), pp. 285–291. ISSN: 1120-009X. DOI: 10.1179/1973947812Y.0000000037. URL: https://www.tandfonline.com/action/journalInformation?journalCode=yjoc20%20http://www.tandfonline.com/doi/full/10.1179/1973947812Y.0000000037.

[83] Nili Schamroth Pravda et al. “Non-Invasive Hemodynamic Whole-Body Bioimpedance Indices for the Early Detection of Cancer Treatment-Related Cardiotoxicity: A Retrospective Observational Study”. In: *Cardiology* 145.6 (2020), pp. 350–355. ISSN: 0008-6312. DOI: 10.1159/000505809. URL: https://www.karger.com/Article/FullText/505809.

[84] Mehmet Ali Nahit Şendur et al. “Comparison of the long term cardiac effects associated with 9 and 52 weeks of trastuzumab in HER2-positive early breast cancer”. In: *Current Medical Research and Opinion* 31.3 (Mar. 2015), pp. 547–556. ISSN: 0300-7995. DOI: 10.1185/03007995.2015.1005834. URL: https://www.tandfonline.com/action/journalInformation?journalCode=icmo20%20https://www.tandfonline.com/doi/full/10.1185/03007995.2015.1005834.

[85] Daniel J. Serie et al. “Genome-wide association study of cardiotoxicity in the NCCTG N9831 (Alliance) adjuvant trastuzumab trial”. In: *Pharmacogenetics and Genomics* 27.10 (Oct. 2017), pp. 378–385. ISSN: 1744-6872. DOI: 10.1097/FPC.0000000000000302. URL: /pmc/articles/PMC5581215/%20/pmc/articles/PMC5581215/?report=abstract%20https://www.ncbi.nlm.nih.gov/pmc/articles/PMC5581215/%20https://journals.lww.com/01213011-201710000-00005.

[86] Fabricio Bragança Silva et al. “Effects of treatment with chemotherapy and/or tamoxifen on the biomarkers of cardiac injury and oxidative stress in women with breast cancer”. In: *Medicine* 96.47 (Nov. 2017), e8723. ISSN: 0025-7974. DOI: 10.1097/MD.0000000000008723. URL: http://dx.doi.org/10.1097/MD.0000000000008723%20https://journals.lww.com/00005792-201711270-00053.

[87] Dorthe Skovgaard, Philip Hasbak, and Andreas Kjaer. “BNP Predicts Chemotherapy-Related Cardiotoxicity and Death: Comparison with Gated Equilibrium Radionuclide Ventriculography”. In: *PLoS ONE* 9.5 (May 2014). Ed. by Claudio Moretti, e96736. ISSN: 1932-6203. DOI: 10.1371/journal.pone.0096736. URL: https://dx.plos.org/10.1371/journal.pone.0096736.

[88] Agnieszka Słowik et al. “Anthracycline-induced cardiotoxicity prevention with angiotensin-converting enzyme inhibitor ramipril in women with low-risk breast cancer: results of a prospective randomized study”. In: *Kardiologia Polska* 78.2 (Feb. 2020), pp. 131–137. ISSN: 1897-4279. DOI: 10.33963/KP.15163. URL: https://journals.viamedica.pl/kardiologia_polska/article/view/82465.

[89] Sasha E. Stanton et al. “Pro1170 Ala polymorphism in HER2-neu is associated with risk of trastuzumab cardiotoxicity”. In: *BMC Cancer* 15.1 (Dec. 2015), p. 267. ISSN: 1471-2407. DOI: 10.1186/s12885-015-1298-6. URL: https://bmccancer.biomedcentral.com/articles/10.1186/s12885-015-1298-6%20http://bmccancer.biomedcentral.com/articles/10.1186/s12885-015-1298-6.

[90] Noriaki Tabata et al. “Outcome of current and history of cancer on the risk of cardiovascular events following percutaneous coronary intervention: a Kumamoto University Malignancy and Atherosclerosis (KUMA) study”. In: *European Heart Journal - Quality of Care and Clinical Outcomes* 4.4 (Oct. 2018), pp. 290–300. ISSN: 2058-5225. DOI: 10.1093/ehjqcco/qcx047. URL: http://www.kumadai-junnai.com/home/wp-content/uploads/akusei.pdf%20https://academic.oup.com/ehjqcco/article/4/4/290/4683474.

[91] Valentina K Todorova et al. “Biomarkers for Presymptomatic Doxorubicin-Induced Cardiotoxicity in Breast Cancer Patients”. In: *PLOS ONE* 11.8 (Aug. 2016). Ed. by William B. Coleman, e0160224. ISSN: 1932-6203. DOI: 10.1371/journal.pone.0160224. URL: https://dx.plos.org/10.1371/journal.pone.0160224.

[92] Valentina K Todorova et al. “Polymorphic Variations Associated With Doxorubicin-Induced Cardiotoxicity in Breast Cancer Patients”. In: *Oncology Research Featuring Preclinical and Clinical Cancer Therapeutics* 25.8 (Sept. 2017), pp. 1223–1229. ISSN: 0965-0407. DOI: 10.3727/096504017X14876245096439. URL: http://www.ingentaconnect.com/content/10.3727/096504017X14876245096439.

[93] Jasper Tromp et al. “Long-term survivors of early breast cancer treated with chemotherapy are characterized by a pro-inflammatory biomarker profile compared to matched controls”. In: *European Journal of Heart Failure* 22.7 (July 2020), pp. 1239–1246. ISSN: 1388-9842. DOI: 10.1002/ejhf.1758. URL: https://onlinelibrary.wiley.com/doi/10.1002/ejhf.1758.

[94] E. Tzolos et al. "Dynamic Changes in High-Sensitivity Cardiac Troponin I in Response to Anthracycline-Based Chemotherapy". In: *Clinical Oncology* 32.5 (May 2020), pp. 292–297. ISSN: 09366555. DOI: 10.1016/j.clon.2019.11.008. URL: https://reader.elsevier.com/reader/sd/pii/S0936655519304960.

[95] Pedro Veronese et al. "Effects of anthracycline, cyclophosphamide and taxane chemotherapy on QTc measurements in patients with breast cancer". In: *PLOS ONE* 13.5 (May 2018). Ed. by Claudio M. Costa-Neto, e0196763. ISSN: 1932-6203. DOI: 10.1371/journal.pone.0196763. URL: https://dx.plos.org/10.1371/journal.pone.0196763.

[96] Christof Vulsteke et al. "Clinical and genetic risk factors for epirubicin-induced cardiac toxicity in early breast cancer patients". In: *Breast Cancer Research and Treatment* 152.1 (July 2015), pp. 67–76. ISSN: 0167-6806. DOI: 10.1007/s10549-015-3437-9. URL: http://link.springer.com/10.1007/s10549-015-3437-9.

[97] Jun Wang et al. "Single-cell RNA sequencing reveals novel gene expression signatures of trastuzumab treatment in HER2+ breast cancer". In: *Medicine* 98.26 (June 2019), e15872. ISSN: 0025-7974. DOI: 10.1097/MD.0000000000015872. URL: http://dx.doi.org/10.1097/MD.0000000000015872%20https://journals.lww.com/00005792-201906280-00006.

[98] Hiromi Watanabe et al. "Congestive Heart Failure During Osimertinib Treatment for Epidermal Growth Factor Receptor (EGFR)-mutant Non-small Cell Lung Cancer (NSCLC)". In: *Internal Medicine* 56.16 (Aug. 2017), pp. 2195–2197. ISSN: 0918-2918. DOI: 10.2169/internalmedicine.8344-16. URL: http://internmed.jp%20https://www.jstage.jst.go.jp/article/internalmedicine/56/16/56_8344-16/_article.

[99] Quinn S. Wells et al. "Genome-wide association and pathway analysis of left ventricular function after anthracycline exposure in adults". In: *Pharmacogenetics and Genomics* 27.7 (July 2017), pp. 247–254. ISSN: 1744-6872. DOI: 10.1097/FPC.0000000000000284. URL: https://pubmed.ncbi.nlm.nih.gov/28542097/%20https://journals.lww.com/01213011-201707000-00001.

[100] Siegfried Wieshammer et al. "Cardiotoxicity and Cancer Therapy: Treatment-Related Cardiac Morbidity in Patients Presenting with Symptoms Suggestive of Heart or Lung Disease". In: *Oncology* 85.3 (2013), pp. 137–144. ISSN: 1423-0232. DOI: 10.1159/000354299. URL: www.karger.com/ocl%20https://www.karger.com/Article/FullText/354299.

[101] Leszek Wojnowski et al. "NAD(P)H Oxidase and Multidrug Resistance Protein Genetic Polymorphisms Are Associated With Doxorubicin-Induced Cardiotoxicity". In: *Circulation* 112.24 (Dec. 2005), pp. 3754–3762. ISSN: 0009-7322. DOI: 10.1161/CIRCULATIONAHA.105.576850. URL: https://www.ahajournals.org/doi/abs/10.1161/circulationaha.105.576850%20https://www.ahajournals.org/doi/10.1161/CIRCULATIONAHA.105.576850.

[102] Wang Yadi et al. "Bioinformatic analysis of peripheral blood miRNA of breast cancer patients in relation with anthracycline cardiotoxicity". In: *BMC Cardiovascular Disorders* 20.1 (Dec. 2020), p. 43. ISSN: 1471-2261. DOI: 10.1186/s12872-020-01346-y. URL: https://bmccardiovascdisord.biomedcentral.com/articles/10.1186/s12872-020-01346-y.

[103] Li-Rong Yu et al. "Immune response proteins as predictive biomarkers of doxorubicin-induced cardiotoxicity in breast cancer patients". In: *Experimental Biology and Medicine* 243.3 (Feb. 2018), pp. 248–255. ISSN: 1535-3702. DOI: 10.1177/1535370217746383. URL: https://www.r-project.org/%20http://journals.sagepub.com/doi/10.1177/1535370217746383.

[104] Dimitrios Zardavas et al. "Role of Troponins I and T and N -Terminal Prohormone of Brain Natriuretic Peptide in Monitoring Cardiac Safety of Patients With Early-Stage Human Epidermal Growth Factor Receptor 2–Positive Breast Cancer Receiving Trastuzumab: A Herceptin Adjuvant Study C". In: *Journal of Clinical Oncology* 35.8 (Mar. 2017), pp. 878–884. ISSN: 0732-183X. DOI: 10.1200/JCO.2015.65.7916. URL: https://ascopubs.org/doi/10.1200/JCO.2015.65.7916.

[105] Simone Meneghetti Zatta et al. "Application of Remote Ischemic Preconditioning in Patients Undergoing Chemotherapy with Anthracyclines". In: *Current Enzyme Inhibition* 15.2 (Oct. 2019), pp. 114–119. ISSN: 15734080. DOI: 10.2174/1573408015666190906120640. URL: http://www.eurekaselect.com/174740/article.

[106] Chu-Jie Zhang et al. "Early anthracycline-induced cardiotoxicity monitored by echocardiographic Doppler parameters combined with serum hs-cTnT". In: *Echocardiography* 34.11 (Nov. 2017), pp. 1593–1600. ISSN: 07422822. DOI: 10.1111/echo.13704. URL: https://pubmed.ncbi.nlm.nih.gov/28942608/%20https://onlinelibrary.wiley.com/doi/10.1111/echo.13704.

[107] Fangfang Zhang et al. "Cathepsin B Dependent Cleavage Product of Serum Amyloid A1 Identifies Patients with Chemotherapy-Related Cardiotoxicity". In: *ACS Pharmacology & Translational Science* 2.5 (Oct. 2019), pp. 333–341. ISSN: 2575-9108. DOI: 10.1021/acsptsci.9b00035. URL: https://pubs.acs.org/doi/abs/10.1021/acsptsci.9b00035%20https://pubs.acs.org/doi/10.1021/acsptsci.9b00035.

[108] Ziyuan Zhao et al. "Dysregulated miR1254 and miR579 for cardiotoxicity in patients treated with bevacizumab in colorectal cancer". In: *Tumor Biology* 35.6 (June 2014), pp. 5227–5235. ISSN: 1010-4283. DOI: 10.1007/s13277-014-1679-5. URL: https://pubmed.ncbi.nlm.nih.gov/24515657/%20http://link.springer.com/10.1007/s13277-014-1679-5.

[109] Fang Zhou, Xike Lu, and Xun Zhang. "Serum miR-30c Level Predicted Cardiotoxicity in Non-small Cell Lung Cancer Patients Treated with Bevacizumab". In: *Cardiovascular Toxicology* 18.3 (June 2018), pp. 284–289. ISSN: 1530-7905. DOI: 10.1007/s12012-018-9457-z. URL: http://link.springer.com/10.1007/s12012-018-9457-z.

[110] Yao Zhu et al. "Intra-individual variability of total cholesterol is associated with cardiovascular disease mortality: A cohort study". In: *Nutrition, Metabolism and Cardiovascular Diseases* 29.11 (Nov. 2019), pp. 1205–1213. ISSN: 09394753. DOI: 10.1016/j.numecd.2019.07.007. URL: https://linkinghub.elsevier.com/retrieve/pii/S0939475319302728.

[57],[69],[92],[88],[56],[54],[75],[10],[19],[26],[44],[105],[98],[18],[77],[67],[15],[24],[25],[74],[95],[91],[87],[63],[104],[12],[72],[7],[5],[48],[30],[89],[102],[52],[47],[84],[82],[103],[70],[45],[23],[37],[13],[42],[101],[8],[14],[83],[11],[100],[33],[60],[50],[3],[16],[106],[20],[97],[86],[85],[99],[35],[39],[90],[29],[80],[73],[38],[107],[76],[110],[43],[17],[2],[28],[53],[51],[66],[40],[71],[31],[64],[65],[94],[55],[36],[34],[79],[6],[27],[61],[21],[108],[49],[59],[4],[46],[109],[32],[58],[22],[81],[62],[96],[78],[68],[41],[1],[93],[9]

Supplementary Table 1: Search strategies used to retrieved the articles from bibliographic databases.

Database	Field	Query
Pubmed	onco-cardiology	(onco-cardiology[All Fields] OR oncocardiology[All Fields]) AND (”gene”[All Fields] OR ”target”[All Fields] OR ”protein”[All Fields] OR ”biomarker”[All Fields] OR ”RNA”[All Fields] OR ”methylation”[All Fields]) NOT (”review”[Publication Type] OR ”review”[Title/Abstract]) AND English[lang] NOT (”in vitro”[All Fields] OR ”in vivo”[All Fields] OR ”rats”[All Fields] OR ”mice”[All Fields] OR ”cell line”[All Fields])
	cardio-oncology	(cardio-oncology[All Fields] OR cardiooncology[All Fields]) AND (”gene”[All Fields] OR ”target”[All Fields] OR ”protein”[All Fields] OR ”biomarker”[All Fields] OR ”RNA”[All Fields] OR ”methylation”[All Fields]) NOT (”review”[Publication Type] OR ”review”[Title/Abstract]) AND English[lang] NOT (”in vitro”[All Fields] OR ”in vivo”[All Fields] OR ”rats”[All Fields] OR ”mice”[All Fields] OR ”cell line”[All Fields])
	oncology AND cardiotoxicity	(”oncology”[All Fields] OR ”cancer”[All Fields]) AND (”cardiotoxicity”[All Fields] OR ”cancer therapeutics-related cardiac dysfunction” [All Fields]) AND (”gene”[All Fields] OR ”target”[All Fields] OR ”protein”[All Fields] OR ”biomarker”[All Fields] OR ”RNA”[All Fields] OR ”methylation”[All Fields]) NOT (”review”[Publication Type] OR ”review”[Title/Abstract]) AND English[lang] NOT (”in vitro”[All Fields] OR ”in vivo”[All Fields] OR ”rats”[All Fields] OR ”mice”[All Fields] OR ”cell line”[All Fields])
	Cancer AND Cardio	(”oncology”[Title/Abstract] OR ”cancer”[Title/Abstract]) AND (”cardio”[Title/Abstract] OR ”atherosclerosis”[Title/Abstract]) AND (”gene”[All Fields] OR ”target”[All Fields] OR ”protein”[All Fields] OR ”biomarker”[All Fields] OR ”RNA”[All Fields] OR ”methylation”[All Fields]) NOT (”review”[Publication Type] OR ”review”[Title/Abstract]) AND English[lang] NOT (”in vitro”[All Fields] OR ”in vivo”[All Fields] OR ”rats”[All Fields] OR ”mice”[All Fields] OR ”cell line”[All Fields])
WoS	Onco-cardiology	SU=”Life Sciences & Biomedicine” AND TS=(onco-cardiology OR oncocardiology) AND TS=(gene OR target OR protein OR RNA OR biomarker OR methylation OR pathway) NOT SO=”review” NOT SO=”letter” NOT SO=”correspondence as topic” NOT SO=”comments” NOT SO=”opinion” NOT TS=(”in vitro” OR ”in vivo” OR rats OR mice OR “cell line”)
	Cardio-oncology	SU=”Life Sciences & Biomedicine” AND TS= (cardio-oncology OR cardiooncology) AND TS=(gene OR target OR protein OR RNA OR biomarker OR methylation OR pathway) NOT SO=”review” NOT SO=”letter” NOT SO=”correspondence as topic” NOT SO=”comments” NOT SO=”opinion” NOT TS=(”in vitro” OR ”in vivo” OR rats OR mice OR “cell line”)
	Onco AND cardiotoxicity	SU=”Life Sciences & Biomedicine” AND TS=(cancer OR oncology) AND TS=(cardiotoxicity OR “cancer therapeutics related cardiac dysfunction”) AND TS=(gene OR target OR protein OR RNA OR biomarker OR methylation OR pathway) NOT SO=”review” NOT SO=”letter” NOT SO=”correspondence as topic” NOT SO=”comments” NOT SO=”opinion” NOT TS=(”in vitro” OR ”in vivo” OR rats OR mice OR “cell line”)
	Onco AND Cardio	SU=”Life Sciences & Biomedicine” AND TS=(”cancer” OR oncology) AND TS=(cardio OR ”atherosclerosis”) AND TS=(gene OR target OR protein OR RNA OR biomarker OR methylation OR pathway) NOT SO=”review” NOT SO=”letter” NOT SO=”correspondence as topic” NOT SO=”comments” NOT SO=”opinion” NOT TS=(”in vitro” OR ”in vivo” OR rats OR mice OR “cell line”)

Database	Field	Query
Scopus	Onco-cardiology	TITLE-ABS(_onco-cardiology_ OR _oncocardiology_) AND (_gene_ OR _target_ OR _protein_ OR _rna_ OR _biomarker_ OR _methylation_ OR _pathway_) AND NOT (_”in vitro”_ OR _”in vivo”_ OR _rat_ OR _mice_ OR _”cell line*”_ OR _culture_) AND (LIMIT-TO(DOCTYPE , _”ar”_)) AND (LIMIT-TO(LANGUAGE , ”English”))
	Cardio-oncology	TITLE-ABS(_cardio-oncology_ OR _cardiooncology_) AND (_gene_ OR _target_ OR _protein_ OR _rna_ OR _biomarker_ OR _methylation_ OR _pathway_) AND NOT (_”in vitro”_ OR _”in vivo”_ OR _rat_ OR _mice_ OR _”cell line*”_ OR _culture_) AND (LIMIT-TO(DOCTYPE , _”ar”_)) AND (LIMIT-TO(LANGUAGE , ”English”))
	Onco AND cardiotoxicity	TITLE-ABS(_onco_ OR _cancer_) AND TITLE-ABS(_cardiotoxicity_ OR “_cancer therapeutics-related cardiac dysfunction_”) AND (_gene_ OR _target_ OR _protein_ OR _rna_ OR _biomarker_ OR _methylation_ OR _pathway_) AND NOT (_”in vitro”_ OR _”in vivo”_ OR _rat_ OR _mice_ OR _”cell line*”_ OR _culture_) AND (LIMIT-TO(DOCTYPE , _”ar”_)) AND (LIMIT-TO(LANGUAGE , ”English”))
	Onco AND Cardio	TITLE-ABS(oncology OR cancer) AND TITLE-ABS(cardio OR atherosclerosis) AND (gene OR target OR protein OR rna OR biomarker OR methylation OR pathway) AND NOT (”in vitro” OR ”in vivo” OR rat OR mice OR ”cell line*” OR culture) AND (LIMIT-TO(DOCTYPE, ”ar”)) AND (LIMIT-TO (LANGUAGE, ”glish”))
Embase	Onco-cardiology	(’onco-cardiology’:ab,ti OR ’oncocardiology’:ab,ti) AND (’protein’ OR ’gene’ OR ’rna’ OR ’methylation’ OR ’biomarker’ OR ’pathway’) NOT (’letter:it’ OR ’editor:it’ OR ’hypothesis:it’ OR comment:it OR opinion:it OR review:it) NOT (’in vitro study’ OR ’in vivo study’ OR ’mouse’ OR ’mice’ OR ’rat’ OR ’cell$ line$’ OR culture) AND [article]/lim AND [humans]/lim AND [embase]/lim
	Cardio-oncology	(’cardio-oncology’:ab,ti OR ’cardiooncology’:ab,ti) AND (’protein’ OR ’gene’ OR ’rna’ OR ’methylation’ OR ’biomarker’ OR ’pathway’) NOT (’letter:it’ OR ’editor:it’ OR ’hypothesis:it’ OR comment:it OR opinion:it OR review:it) NOT (’in vitro study’ OR ’in vivo study’ OR ’mouse’ OR ’mice’ OR ’rat’ OR ’cell$ line$’ OR culture) AND [article]/lim AND [humans]/lim AND [embase]/lim
	Onco AND cardiotoxicity	(’cancer’:ab,ti OR ’oncology’:ab,ti) AND (’cardiotoxicity’:ab,ti OR ’cancer therapeutics related cardiac dysfunction’:ab,ti) AND (’protein’ OR ’gene’ OR ’rna’ OR ’methylation’ OR ’biomarker’ OR ’pathway’) NOT (’letter:it’ OR ’editor:it’ OR ’hypothesis:it’ OR comment:it OR opinion:it OR review:it) NOT (’in vitro study’ OR ’in vivo study’ OR ’mouse’ OR ’mice’ OR ’rat’ OR ’cell line’ OR culture) AND [article]/lim AND [humans]/lim AND [embase]/lim
	Onco AND Cardio	(’cancer’:ab,ti OR ’oncology’:ab,ti) AND (’cardio’:ab,ti OR ’atherosclerosis’:ab,ti) AND (’protein’ OR ’gene’ OR ’rna’ OR ’methylation’ OR ’biomarker’ OR ’pathway’) NOT (’letter:it’ OR ’editor:it’ OR ’hypothesis:it’ OR comment:it OR opinion:it OR review:it) NOT (’in vitro study’ OR ’in vivo study’ OR ’mouse’ OR ’mice’ OR ’rat’ OR ’cell$ line$’ OR culture) AND [article]/lim AND [humans]/lim AND [embase]/lim

Objects and fields used in the OncoCardio database

Supplementary material 2 to the paper "OncoCardioDB: A Public and Curated Database of Molecular Information in Onco-cardiology/Cardio-oncology"

A.L. Riffo-Campos, J Domingo, E. Dura
(angela.riffo@ufrontera.cl, Juan.Domingo@uv.es, Esther.Dura@uv.es)

Abstract

This document is an explanation of the terms (objects and fields) used for the construction and internal organization of a database for onco-cardiology and their associated genes. Also, it explains some details about data curation and choice of standards. It is delivered as appendix/additional material of the paper about the design, implementation and population of such database.

1 Data curation

The information to fill this database comes essentially from two types of sources: lists of items or standard classifications of items already available and concrete biomedical studies done on a population of human subjects. In our case, the lists and standards are Entrez for genes, International Classification of Diseases (ICD10) for pathologies and Anatomical Therapeutic Chemical Classification (ATC) for drugs. On the other hand, relevant fields of each medical study are collected from the medical literature. Therefore, some information does not depend on the consulted studies, neither it is specific for onco-cardiology. It is to be stored in tables that from now on will be called generic tables. These are:

- Tables 'Gene', 'GeneTranscript', 'Synonym', 'GeneInEnsemble', 'Variation' and 'GenomeFullPosition' as long as those which relate them ('transcribes_as' and 'has_ensemble_id') were filled from the information provided by the R package `org.Hs.eg.db` included in Bioconductor ([1, 3]). This time a R script instead of a Perl macro was used to generate the SQL sentences.
- Table 'Drug' was filled from the classification of the Anatomical Therapeutic Chemical classification (ATC) as provided by the WHO Collaborating Centre for Drug Statistics Methodology of Norway ([4]) first, extracting it as an ASCII text file and then processing it with a Perl macro.
- Table 'Pathology' was filled from the International Classification of Diseases (ICD10) as provided by the Web's Free 2022 ICD-10-CM/PCS Medical Coding Reference ([2]). Again, the information was obtained as an ASCII table and processed by a Perl macro.

The rest of the tables were populated from the fields present in the spreadsheet filled from the published studies; they will be called specific tables. As stated before, such spreadsheet was filled by experts in the area. This time the Perl macro makes a careful check before generating the SQL sentences. Indeed, a list of constraints must be met:

- Values for numeric fields are really numbers and they are in the appropriate interval.
- Strings of characters do not exceed the intended lengths, as designed in the database tables.
- Unknown values are stored always with the same mark.
- Dates follow a standardized format (yyyy-mm-dd).
- Country codes are taken for the known list of countries (ISO country codes).
- Some fields, namely therapies, detection methods and validation methods must belong to a predefined list.

- Statistical significance must be provided as one of a predefined list of known possibilities for its meaning.
- Values for items already stored in the previously filled generic tables (i.e.: all those related with genes, drugs and pathologies) really correspond to an existing value.
- No value is introduced as a duplicate row (for instance: panel kit names are incorporated only when they are mentioned for the first time, as long as drug codes, pathology codes, etc).

2 Naming conventions

The following conventions have been used:

- Tables that represent objects (entities) will be named after the name of the entity, first letter in upper case. The different words in the name (and, in general, in all names of fields) will be capitalized.
- The primary key for each table will in most cases be called 'IdName', being Name the name of the object (table), with a few exceptions.
- The foreign keys will be named as the name of the key they refer to.
- There are two reasons by which a key cannot be NULL: either we know it represents important information that should not be omitted (marked as 'not NULL by choice') or it is a foreign key which refers to the primary key of another table (marked as 'not NULL by design').
- Tables that represent relationships between objects (arising from an N:M cardinality) will have a significant name all in lower case with words separated by the underscore character.
- Fields containing a Digital Object Identifier (DOI) representing different publications will be stored as URLs in the form https://doi.org/<the_doi>

With respect to gene denotation, we have used the R Bioconductor package org.Hs.eg.db which according to its documentation is primarily based on mapping using Entrez Gene identifiers. Therefore, we have used the Entrez identifier as primary key, since it is unique in this database. Also, analyzing it we can observe that each of the (currently) 61547 different Entrez id's has exactly one gene symbol associated, even though a few symbols are associated to two different Entrez id's (and even one it is associated to three Entrez id's). With respect to Ensembl identifiers, many Entrez id's have no an associated Ensembl id and some Ensemble identifiers have more than one associated Entrez. This has forced the introduction of a new table to store genes by their Ensemble identifiers and relate it with the first gene table with a suitable relation. The same situation happens between Entrez identifiers and transcripts which gives rise to a secondary relation table, too.

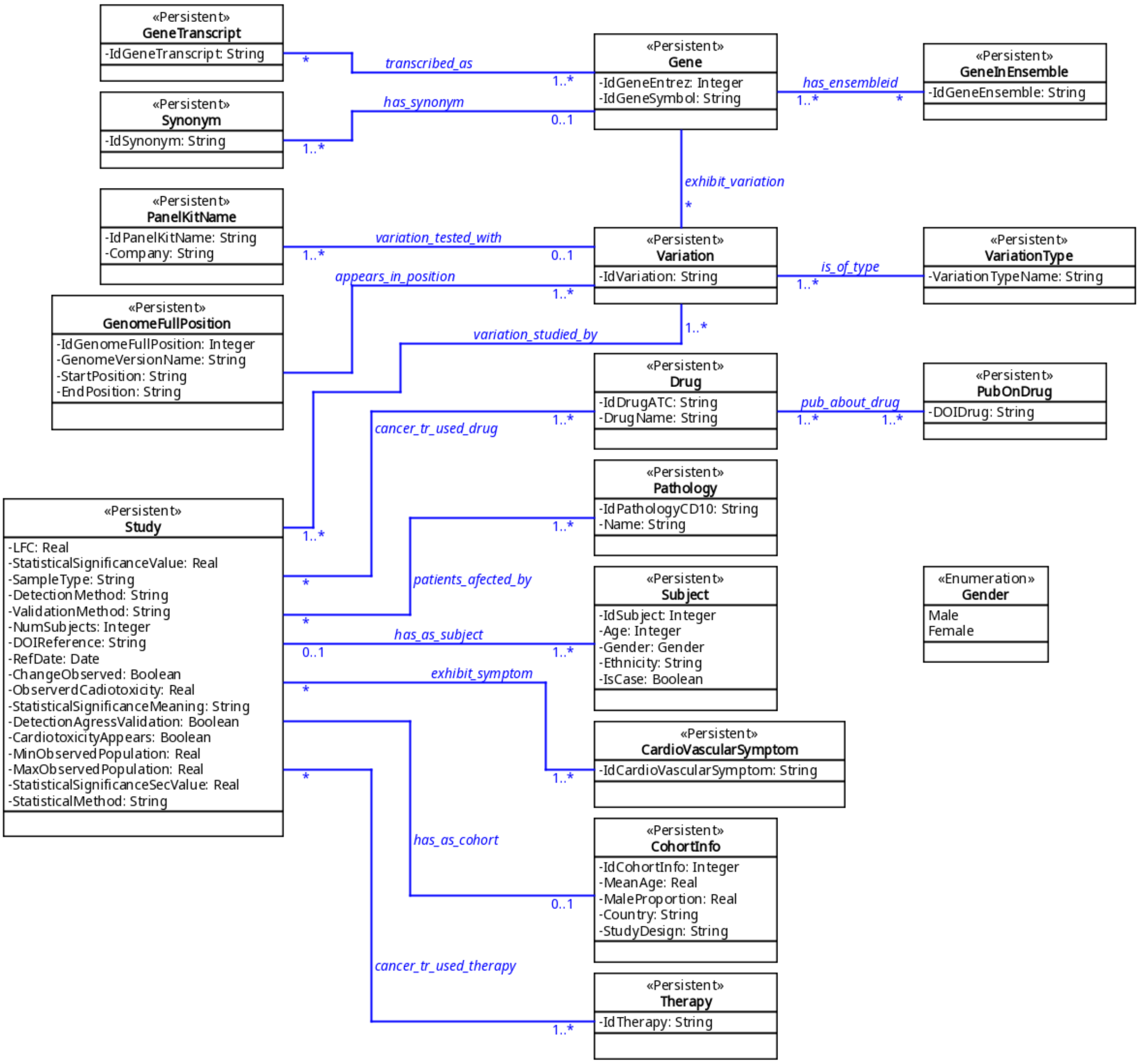

Figure 1: UML diagram of the database.

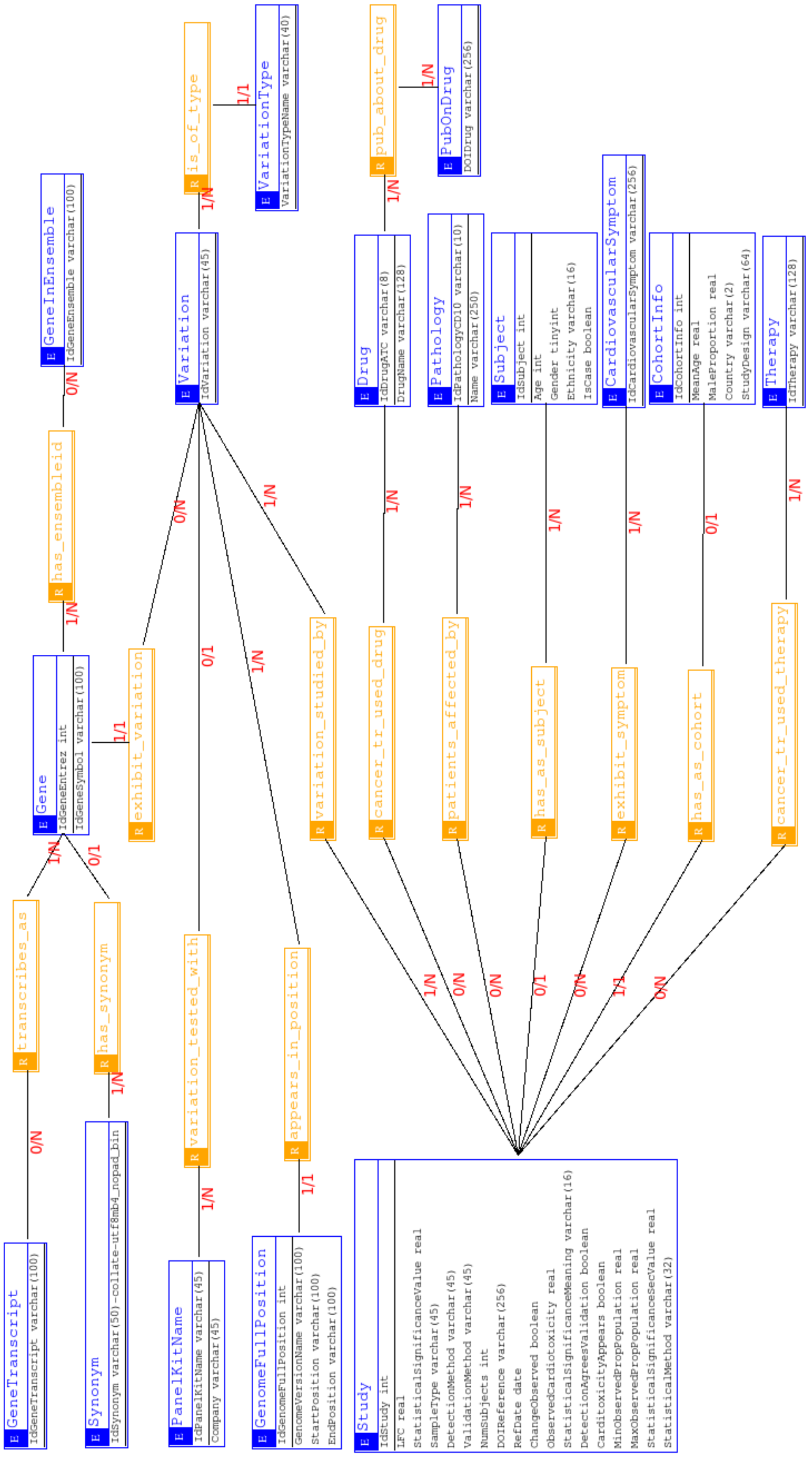

Figure 2: Proposed entity/relationship model.

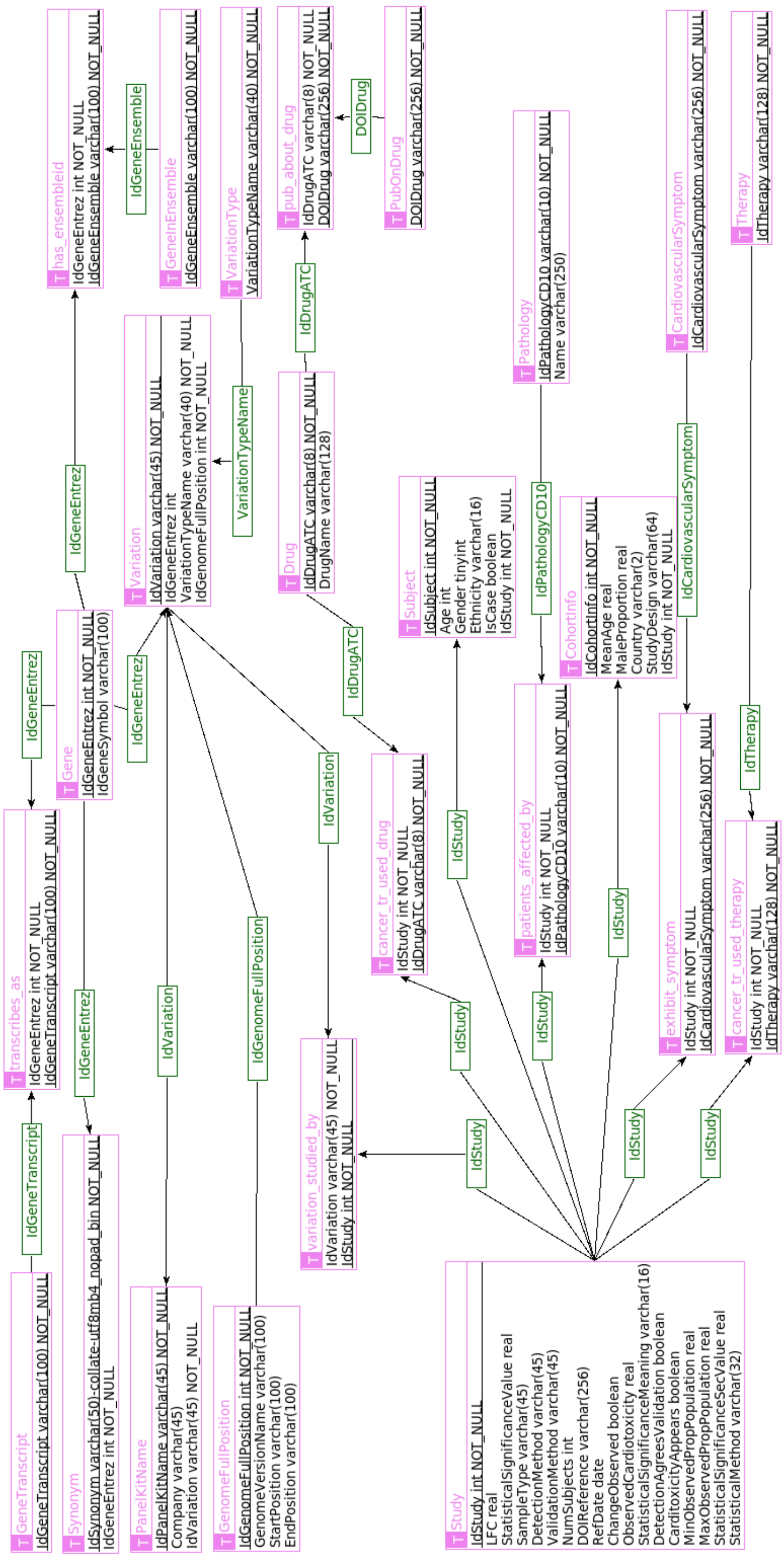

Figure 3: Tables of the database generated by the E/R model.

3 Tables corresponding to entities

Object: **Gene**

Identifier	Type	Meaning
IdGeneEntrez<br>Primary key (**NOT** NULL).	`INT`	The gene identifier in the Entrez gene database (see Entrez).
IdGeneSymbol<br>**NOT** NULL by choice.	`VARCHAR(100)`	The symbol used in the genetic notation for the gene. This field will always have a value since it has been tested that every Entrez identifier has an associated symbol.

Object: **GeneInEnsemble**

Identifier	Type	Meaning
IdGeneEnsemble<br>Primary key (**NOT** NULL).	`VARCHAR(100)`	The gene identifier in the Ensemble gene database (see Ensemble).

Object: **GeneTranscript**

Identifier	Type	Meaning
IdGeneTranscript<br>Primary key (**NOT** NULL).	`VARCHAR(100)`	The identifier of this transcript. It uses to be similar to the Ensemble designation for a gene, but with some letters changed and/or added.

Object: **Synonym**

Identifier	Type	Meaning
IdSynonym<br>Primary key (**NOT** NULL).	`VARCHAR(50)`	The name of the synonym.
IdGeneEntrez<br>**NOT** NULL by design.	`INT`	A reference to the Entrez id of the gene of which this object is a synonym. Notice that several synonyms can designate the same gene (i.e.: the same Entrez id) so this field may take repeated values in several rows.

Object: **Variation**

Identifier	Type	Meaning
IdVariation<br>Primary key (**NOT** NULL).	`VARCHAR(45)`	The name used to designate this variation. It is standard, depending on the nature of the variation.
IdGeneEntrez<br>Can be NULL	`INT`	The reference to the primary key of the gene this variation refers to. We assume that a variation manifests itself in only one gene, but it might be an intergenic variation, too, not associated to a particular gene. In that case this field would be NULL.

VariationTypeName<br>**NOT** NULL by design.	`VARCHAR(40)`	The reference to the variation type this variation belongs to.
IdGenomeFullPosition<br>**NOT** NULL by design.	`INT`	The reference to the GenomeFullPosition object that contains the position in the genome at which this variation occurs.

Object: **VariationType**

Identifier	Type	Meaning
VariationTypeName<br>Primary key (**NOT** NULL).	`VARCHAR(40)`	The type of the variation. Variants have a type which refers to its role in biological processes. Currently, 11 types are admitted: SNV, SNP, InDel, CNV, abundance proteins, differential expression, DNA methylation, RNA methylation, histone modifications, Post-translational modification and metabolites. Others could be added, if needed.

Object: **PanelKitName**

Identifier	Type	Meaning
IdPanelKitName<br>Primary key (**NOT** NULL).	`VARCHAR(45)`	The name of the panel kit, as given by the company that manufactures it.
Company<br>**NOT** NULL by choice.	`VARCHAR(45)`	The name of the company that manufactures this panel.
IdVariation<br>**NOT** NULL by design.	`VARCHAR(45)`	The identifier of the variation whose expression is tested with this panel kit.

Object: **GenomeFullPosition**

Identifier	Type	Meaning
IdGnomeFullPosition<br>Primary key (**NOT** NULL).	`INT`	A unique identifier for this position object. Unfortunately, it has to be arbitrary, since this table needs a primary key and none of its components can play such role.
GenomeVersionName<br>**NOT** NULL by choice.	`VARCHAR(100)`	The name of this genome version, like GRCh37, as obtained either from USC or from Ensembl. There are consortia that update the human genome regularly.
StartPosition<br>**NOT** NULL by choice.	`VARCHAR(100)`	The place in the sequence where this genome version starts in the version of reference genome expressed in the former field, like chr6:76,573,879.
EndPosition<br>**NOT** NULL by choice.	`VARCHAR(100)`	The place in the sequence where this genome version ends in the version of reference genome expressed in the former field, as before.

Object: **Pathology**

Identifier	Type	Meaning
IdPathologyCD10<br>Primary key (**NOT** NULL).	`VARCHAR(10)`	The identifier for the pathology, as given by the ICD10 classification of 2021 (see IDC10).
Name<br>**NOT** NULL by choice.	`VARCHAR(200)`	The CD10 name of the pathology. All codes in the CD10 table have a name, so this field will not be NULL. It can be ambiguous, so pathologies should be primarily identified by their CD10 codes.

Object: **Drug**

Identifier	Type	Meaning
IdDrugATC<br>Primary key (**NOT** NULL).	`VARCHAR(8)`	A unique identifier for the drug according to the ATC (Anatomical Therapeutic Chemical Classification System). See ATC.
DrugName<br>**NOT** NULL by choice.	`VARCHAR(128)`	The ATC drug name. All codes in the ATC table have a name, so this field will not be NULL. It can be ambiguous, so drugs should be primarily identified by their ATC codes.

Object: **Study**

Identifier	Type	Meaning
IdStudy<br>Primary key (**NOT** NULL).	`INT`	A unique arbitrary identifier for an study. Differently to the other primary keys, this is completely arbitrary, since there is no standard or accepted way to give something like a “reference number” to the studies a research group decides to make.
StatisticalMethod<br>**NOT** NULL by choice.	`VARCHAR(32)`	It refers to the statistical method with which the ”StatisticalSignificanceMeaning” is obtained. For instance, t-test, Cox elastic net regression, general linear model, etc. When the method is not specified, the field takes as value ”unspecified”.
StatisticalSignificanceMeaning<br>**NOT** NULL by choice.	`VARCHAR(16)`	The way in which statistical significance of the experiment is expressed. It can only be one of these strings: BH (also incorrectly denoted as FDR), Bonferroni, OddsRatio or pVal.
StatisticalSignificanceValue<br>**NOT** NULL by choice.	`REAL`	The value of the significance, expressed as stated in the field ‘StatisticalSignificanceMeaning”.
SampleType<br>**NOT** NULL by choice.	`VARCHAR(45)`	The type of sample, such as blood, tissue, plaque, saliva, etc.
DetectionMethod<br>**NOT** NULL by choice.	`VARCHAR(45)`	The detection method used. There includes all the massive ones like arrays and sequencing and also the specific ones like PCR, etc. as long as new methods when available.
ValidationMethod<br>Can be NULL	`VARCHAR(45)`	The validation method used, that is, if a different technique is used to confirm that the results will be the same regardless of the biological technique used to obtain the data. If not used, this field takes as value ”None”.
NumSubjects<br>**NOT** NULL by choice.	`INT`	The number of subjects used for this study.
DOIReference<br>**NOT** NULL by choice.	`VARCHAR(256)`	The DOI of the publication that reported this study.<br>**WARNING:** Notice that this publication is not the same as those used in the PubOnDrug table.
RefDate<br>**NOT** NULL by choice.	`DATE`	The date of the publication that reported this study, in format yyyy-mm-dd.

ChangeObserved<br>**NOT** NULL by choice.	`BOOLEAN`	Boolean value to mark if the study has shown any change in the expression of affected cases vs. controls.
CarditoxicityAppears<br>**NOT** NULL by design.	`BOOLEAN`	A mark to indicate if the drug analyzed in this study showed cardiotoxicity effects (TRUE) or not (FALSE).
ObservedCarditoxicity<br>Can be NULL	`REAL`	The proportion (as a number in 0..1) of the total participants who manifested cardiotoxic effects. It can be NULL if either the former field is FALSE (no cardiotoxic effects have been observed) or the study does not give any value for this parameter.
DetectionAgreesValidation<br>Can be NULL	`BOOLEAN`	In the case that a validation of the results (ValidationMethod) has been carried out, it is included if the results were concordant between both biological techniques, taking the value of TRUE. If the result was not in agreement or in the case that a validation has not been carried out, then the field takes the value of FALSE.
MinObservedPropPopulation<br>Can be NULL	`FLOAT`	The lowest value of the proportion of the general population in which the effects associated to this study has been observed. Restriction: must be in [0..1]. NULL value means unknown or unspecified.
MaxObservedPropPopulation<br>Can be NULL	`FLOAT`	The highest value of the proportion of the general population in which the effects associated to this study has been observed. If the available datum is not an interval but a single value both of these fields will have to be filled with the same value. Restriction: must be in [0..1] and must be $\geq$ MinObservedPropPopulation. NULL value means unknown or unspecified.

Object: **PubOnDrug**

Identifier	Type	Meaning
DOIDrug<br>Primary key (**NOT** NULL).	`VARCHAR(256)`	The DOI that points to a significant publication about a drug (the first of most important one about this drug and/or its use in this context).<br>**WARNING:** This is not the same as the field used in the Variation and 'cancer_tr_used_drug' tables. Currently, this table is empty; to be filled in the future, if possible.

Object: **Subject**

Identifier	Type	Meaning
NumSubject<br>Primary key (**NOT** NULL).	`INT`	An arbitrary integer number to designate each of the subjects that participated in the study.<br>**WARNING:** Currently, this table is empty since studies do not use to provide detailed (disaggregated) information about each subject of the cohort. Nevertheless, is kept for the case any study would have done or would do it.
Age<br>Can be NULL	`INT`	The age of this subject.
Gender<br>Can be NULL	`TINYINT`	A number to identify the gender of this subject.
Ethnicity<br>Can be NULL	`VARCHAR(16)`	An identifier of the race of this subject.

IsCase<br>Can be NULL	`BOOLEAN`	A mark to indicate if this subject participated in the study as case (TRUE) or control (FALSE).
IdStudy<br>**NOT** NULL by design.	`INT`	A reference to the identifier of the study this subject was part of.

Object: **CohortInfo**

Identifier	Type	Meaning
IdCohortInfo<br>Primary key (**NOT** NULL).	`INT`	An arbitrary integer number to designate each of the cohorts associated to any study.
MeanAge<br>Can be NULL	`REAL`	The mean age of the subjects who participated in the study.
MaleProportion<br>Can be NULL	`REAL`	The proportion (as a number in 0..1) of the total participants who were males. Female proportion can be obtained as 1 minus this value.
StudyDesign<br>Can be NULL	`VARCHAR(64)`	The study design used for the experiment. This field can take values such as: prospective cohort study, case-control study, etc. If it is not specified in the study, "Not included" is included.
Country<br>Can be NULL	`VARCHAR(2)`	The country in which the study took place, using the international standard for country designation ISO 3166-1
IdStudy<br>**NOT** NULL by design.	`INT`	A reference to the identifier of the study this subject was part of.

Object: **CardiovascularSymptom**

Identifier	Type	Meaning
IdCardiovascularSymptom<br>Primary key (**NOT** NULL).	`VARCHAR(256)`	The clinical denomination of a symptom that can be observed in the cardiovascular system due to the administration of a drug.

Object: **Therapy**

Identifier	Type	Meaning
IdTherapy<br>Primary key (**NOT** NULL).	`VARCHAR(128)`	The name of a therapy used to treat a cancer. It can be one of these: chemotherapy,radiotherapy,targeted therapy,immunotherapy,surgery and hormonal, or any combination of them (so there will be up to 64 possibilities). It can also be none, indicating that no therapy was applied.

4 Tables corresponding to relations between entities

Object: **has_ensembleid**

Identifier	Type	Meaning
IdGeneEntrez<br>Primary key (**NOT** NULL).	`INT`	An Entrez identifier for a gene (points to the homonymous field of table `Gene`).
IdGeneEnsemble<br>Primary key (**NOT** NULL).	`VARCHAR(100)`	An Ensemble identifier for a gene (points to the homonymous field of table `GeneInEnsemble`).

Object: **transcribes_as**

Identifier	Type	Meaning
IdGeneTranscript<br>Primary key (**NOT** NULL).	`VARCHAR(100)`	A reference to the transcript name (points to the homonymous field of table `GeneTranscript`).
IdGeneEntrez<br>**NOT** NULL by design.	`INT`	A reference to the Entrez id of the transcript (points to the homonymous field of table `Gene`).

Object: **pub_about_drug**

Identifier	Type	Meaning
IdDrugATC<br>Primary key (**NOT** NULL).	`VARCHAR(8)`	The identifier of a drug that is mentioned in a publication (points to the homonymous field of table `Drug`).
DOIDRUG<br>Primary key (**NOT** NULL).	`VARCHAR(256)`	The DOI of the publication that first or most importantly mentions the drug whose ATC code is in this table. This relation arises from the fact that the Drugbank database contains multiple publications about each drug, and also a publication might appear in it repeated (i.e.: associated to more than one drug).

Object: **variation_studied_by**

Identifier	Type	Meaning
IdVariation<br>Primary key (**NOT** NULL).	`VARCHAR(45)`	The identifier of a variation that is discovered by a study (points to the homonymous field of table `Variation`).
IdStudy<br>Primary key (**NOT** NULL).	`INT`	The identifier of an study that has discovered a variation (points to the homonymous field of table `Study`).

Object: **patients_affected_by**

Identifier	Type	Meaning
IdStudy<br>Primary key (**NOT** NULL).	`INT`	The identifier of a study that reports the patients to suffer from one or more pathologies (points to the homonymous field of table `Study`).

IdPathologyCD10<br>Primary key (**NOT** NULL).	`VARCHAR(10)`	The ICD10 code of each of the pathologies that the study reports to have been observed in its cohort of subjects (points to the homonymous field of table `Pathology`).

Object: **exhibit_symptom**

Identifier	Type	Meaning
IdStudy<br>Primary key (**NOT** NULL).	`INT`	The identifier of a study that reports the patients to exhibit one or more cardiovascular symptoms (points to the homonymous field of table `Study`).
IdCardiovascularSymptom<br>Primary key (**NOT** NULL).	`VARCHAR(256)`	The identifier of each of the cardiovascular symptoms that the study reports to have been observed in its cohort of subjects (points to the homonymous field of table `CardioVascularSymptom`).

Object: **cancer_tr_used_drug**

Identifier	Type	Meaning
IdStudy<br>Primary key (**NOT** NULL).	`INT`	The identifier of a study that reports that the patients have suffered cancer and have been treated with some drug (points to the homonymous field of table `Study`).
IdDrugATC<br>Primary key (**NOT** NULL).	`VARCHAR(8)`	The ATC identifier of a drug that was used to treat the cancer of the patients in the related study (points to the homonymous field of table `Drug`).

Object: **cancer_tr_used_therapy**

Identifier	Type	Meaning
IdStudy<br>Primary key (**NOT** NULL).	`INT`	The identifier of a study that reports that the patients have suffered cancer and have been treated by the application of some therapy (points to the homonymous field of table `Study`).
IdTherapy<br>Primary key (**NOT** NULL).	`VARCHAR(128)`	The therapy identifier (the therapy name) that was used to treat the cancer of the patients in the related study (points to the homonymous field of table `Therapy`).

References

[1] Marc Carlson. *org.Hs.eg.db: Genome wide annotation for Human.* R package version 3.13.0. 2021.

[2] ICD10Data. *The Web's Free 2022 ICD-10-CM/PCS Medical Coding Reference.* 2021. URL: https://www.icd10data.com/.

[3] Martin Morgan. *BiocManager: Access the Bioconductor Project Package Repository*. R package version 1.30.16. 2021. URL: https://CRAN.R-project.org/package=BiocManager.

[4] WHO Collaborating Centre for Drug Statistics Methodology of Norway. *Guidelines for ATC classification and DDD assignment, 2021. Oslo, 2020*. 2021. URL: https://www.whocc.no/atc_ddd_index/.

Data on content of the OncoCardio database

Supplementary material 3 to the paper "OncoCardioDB: A Public and Curated Database of Molecular Information in Onco-cardiology/Cardio-oncology"

A.L. Riffo-Campos, J Domingo, E. Dura
(angela.riffo@ufrontera.cl, Juan.Domingo@uv.es, Esther.Dura@uv.es)

Total number of Entrez identifiers:

Asked query: SELECT COUNT(IdGeneEntrez) FROM Gene;
Result table:

COUNT)
61548

Number of different Entrez identifiers that appear in any study:

Asked query: SELECT COUNT(DISTINCT Gene.IdGeneEntrez) FROM Gene,Variation,variation_studied_by
WHERE Gene.IdGeneEntrez=Variation.IdGeneEntrez AND variation_studied_by.IdVariation=Variation.IdVariation;
Result table:

COUNT
93

Gene Entrez identifiers that appear and their names ordered by number of occurrences

Asked query: SELECT Gene.IdGeneEntrez,Gene.IdGeneSymbol,COUNT(Gene.IdGeneEntrez) FROM Gene,Variation,variation[illegible]
WHERE Gene.IdGeneEntrez=Variation.IdGeneEntrez AND variation_studied_by.IdVariation=Variation.IdVariation
GROUP BY Gene.IdGeneEntrez ORDER BY COUNT(Gene.IdGeneEntrez) DESC;
Result table:

IdGeneEntrez	IdGeneSymbol	COUNT)
4879	NPPB	20
7139	TNNT2	15
7137	TNNI3	14
1401	CRP	7
2064	ERBB2	4
874	CBR3	3

IdGeneEntrez	IdGeneSymbol	COUNT)
1535	CYBA	3
4689	NCF4	3
5243	ABCB1	3
9518	GDF15	3
534	ATP6V1G2	3
4353	MPO	3
4795	NFKBIL1	3
5447	POR	3
1244	ABCC2	2
2950	GSTP1	2
4049	LTA	2
4363	ABCC1	2
112267902	LOC112267902	2
4439	MSH5	2
5880	RAC2	2
212	ALAS2	1
355	FAS	1
383	ARG1	1
1158	CKM	1
1668	DEFA3	1
2167	FABP4	1
2244	FGB	1
3106	HLA-B	1
3490	IGFBP7	1
3569	IL6	1
4282	MIF	1
4878	NPPA	1
5155	PDGFB	1
5327	PLAT	1
5834	PYGB	1
5919	RARRES2	1
6347	CCL2	1
6647	SOD1	1
7035	TFPI	1
7097	TLR2	1
7124	TNF	1
7364	UGT2B7	1
7799	PRDM2	1
10673	TNFSF13B	1
25794	FSCN2	1
255738	PCSK9	1
406904	MIR1-1	1
406938	MIR146A	1
406967	MIR192	1
406984	MIR200B	1
407040	MIR34A	1
693159	MIR574	1
100126334	MIR885	1
100507436	MICA	1

IdGeneEntrez	IdGeneSymbol	COUNT)
106480086	RNU6-1186P	1
240	ALOX5	1
356	FASLG	1
1088	CEACAM8	1
1241	LTB4R	1
1577	CYP3A5	1
1669	DEFA4	1
2109	ETFB	1
2214	FCGR3A	1
2246	FGF1	1
3084	NRG1	1
3339	HSPG2	1
3497	IGHE	1
4000	LMNA	1
4151	MB	1
5228	PGF	1
5266	PI3	1
6288	SAA1	1
6368	CCL23	1
6696	SPP1	1
7040	TGFB1	1
7099	TLR4	1
7273	TTN	1
7450	VWF	1
9173	IL1RL1	1
10665	TSBP1	1
10850	CCL27	1
58191	CXCL16	1
406885	MIRLET7C	1
406913	MIR126	1
406952	MIR17	1
406982	MIR20A	1
406992	MIR210	1
494327	MIR378A	1
693164	MIR579	1
100507428	LINC00458	1
105377364	LOC105377364	1
107984727	LOC107984727	1

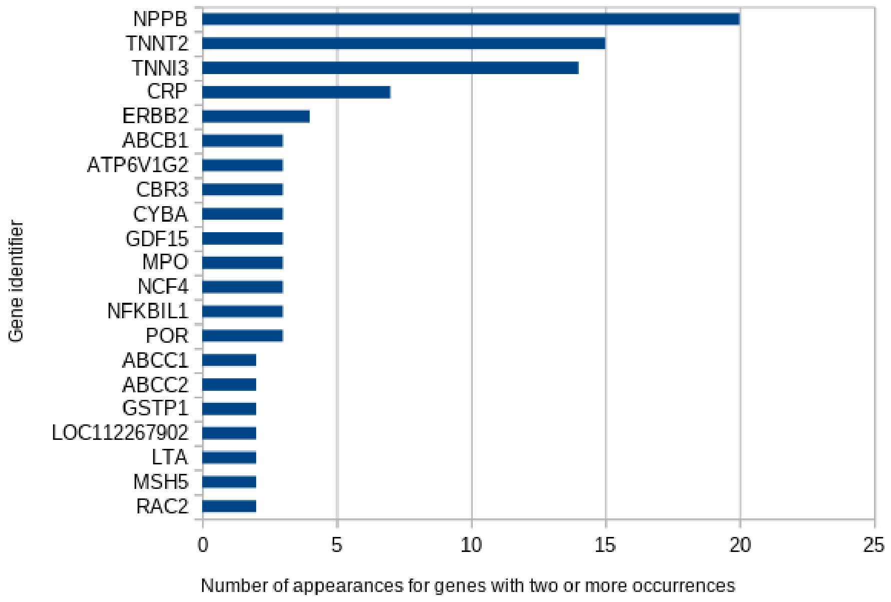

Number of different variations that appear in any study

Asked query: SELECT COUNT(DISTINCT IdVariation) FROM variation_studied_by;
Result table:

COUNT
107

Different variations that appear ordered by number of occurrences

Asked query: SELECT IdVariation, COUNT(IdVariation) from variation_studied_by GROUP BY IdVariation ORDER BY COUNT(IdVariation) DESC;
Result table:

IdVariation	COUNT
NPPB	20
TNNT2	15
TNNI3	14
CRP	7
rs1056892	3
rs4673	3
MPO	3

IdVariation	COUNT
GDF15	3
rs1883112	3
rs1695	2
rs1058808	2
rs13058338	2
rs1136201	2
CCL2	1
CKM	1
DEFA4	1
FGB	1
IGFBP7	1
let-7c	1
miR-146a-5p	1
miR-34a-5p	1
MIR1-1	1
MIR579	1
PCSK9	1
PLAT	1
rs10127939	1
rs11932853	1
rs2050190	1
rs2229109	1
rs2523619	1
rs3130059	1
rs7668258	1
rs8187710	1
SAA1	1
TGFB1	1
ALAS2	1
CCL23	1
FABP4	1
FGF1	1
IGHE	1
LTB4R	1
miR-17-5p	1
MiR-378	1
MIR126	1
PDGFB	1
proANP	1
rs1041981	1
rs17222723	1
rs2071591	1
rs2235047	1
rs28415722	1
rs3131378	1
rs4732513	1
rs776746	1
rs909253	1
SOD1	1

IdVariation	COUNT
TLR2	1
ALOX5	1
CCL27	1
CXCL16	1
FAS	1
IL1RL1	1
MB	1
miR-192-5p	1
miR-574-3p	1
MIR20A	1
PGF	1
PYGB	1
rs1045642	1
rs13240755	1
rs1800629	1
rs2071592	1
rs246221	1
rs2868177	1
rs3131379	1
rs7406710	1
rs79338777	1
rs9264942	1
SPP1	1
TLR4	1
TTN:c.53918del (p.Gly17973fs)	1
ARG1	1
CEACAM8	1
DEFA3	1
FASLG	1
HSPG2	1
IL6	1
MIF	1
miR-200b-3p	1
miR-885-5p	1
MIR210	1
NRG1	1
PI3	1
RARRES2	1
rs10484554	1
rs11796	1
rs1553265328	1
rs2071594	1
rs2523451	1
rs3093949	1
rs45511401	1
rs7542939	1
rs8032978	1
rs9316695	1
TFPI	1

IdVariation	COUNT
TNFSF13B	1
VWF	1

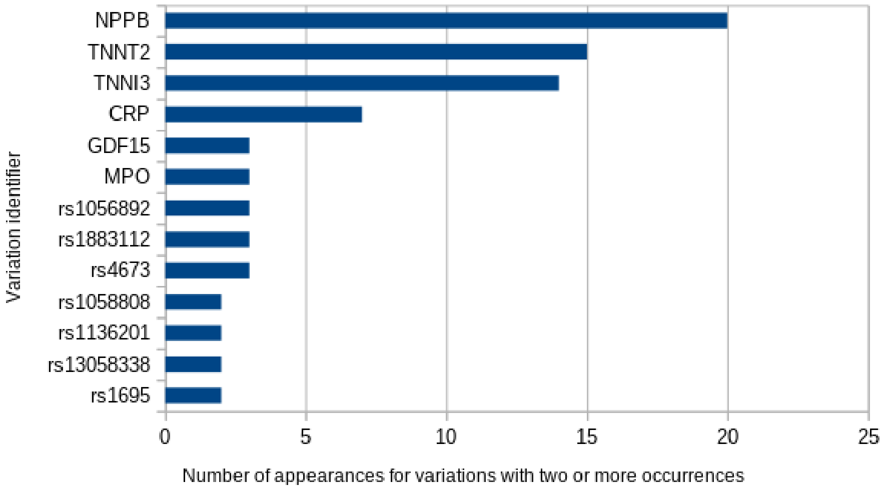

Total number of drugs stored in the database (by its ATC code and name)

Asked query: SELECT COUNT(IdDrugATC) FROM Drug;
Result table:

COUNT
6460

Number of drugs which appear in any study

Asked query: SELECT COUNT(IdDrugATC) FROM cancer_tr_used_drug
Result table:

COUNT
259

Number of different drugs which appear in any study

(This is different from the former one, since some studies mention many drugs).
Asked query: SELECT COUNT(distinct IdDrugATC) FROM cancer_tr_used_drug;
Result table:

COUNT
26

ATC codes and names of the drugs which are mentioned in any study, ordered by number of occurrences

Asked query: SELECT cancer_tr_used_drug.IdDrugATC,Drug.DrugName,COUNT(cancer_tr_used_drug.IdDrugATC) FROM cancer_tr_used_drug,Drug WHERE cancer_tr_used_drug.IdDrugATC=Drug.IdDrugATC GROUP BY cancer_tr_used_drug.IdDrugATC ORDER BY COUNT(cancer_tr_used_drug.IdDrugATC) DESC;
Result table:

IdDrugATC	DrugName	COUNT
L01DB01	doxorubicin	58
L01AA01	cyclophosphamide	35
L01XC03	trastuzumab	32
L01DB	Anthracyclines and related substances	30
L01DB03	epirubicin	24
L01CD02	docetaxel	11
L01BC02	fluorouracil	8
L01XC02	rituximab	8
L01CD01	paclitaxel	8
L01CA02	vincristine	8
L01CD	Taxanes, antineoplastic	7
H02AB07	prednisone	7
L01XA01	cisplatin	4
L01DB02	daunorubicin	3
L04AC07	tocilizumab	2
L04AX03	methotrexate	2
L02BA01	tamoxifen	2
L04AC08	canakinumab	2
L01XE05	sorafenib	1
L01CB01	etoposide	1
L01DB07	mitoxantrone	1
L01AA03	melphalan	1
L01XC07	bevacizumab	1
L01XA02	carboplatin	1
L01XX45	carfilzomib	1
S03BA02	prednisolone	1

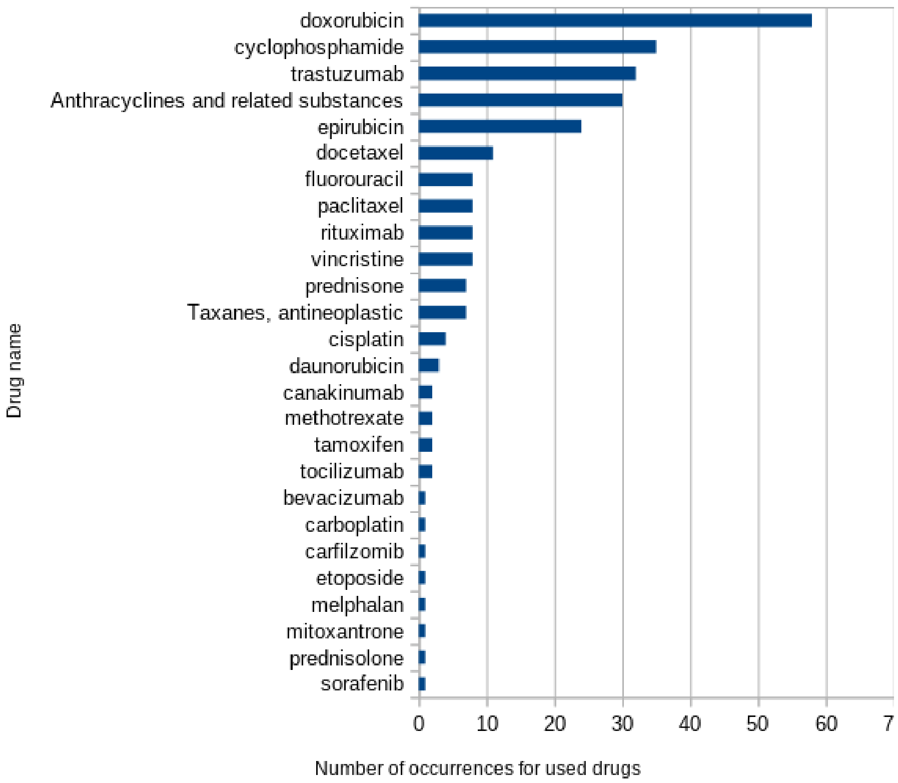

Total number of diseases (pathologies) stored in the data base (by its ICD10 code and name)

Asked query: SELECT COUNT(IdPathologyCD10) FROM Pathology;
Result table:

COUNT
46564

Number of different pathologies mentioned in any study

Asked query: SELECT COUNT(IdPathologyCD10) FROM patients_affected_by;
Result table:

COUNT
237

Pathologies that appear in any study, ordered by its number of occurrences

Asked query: SELECT patients_affected_by.IdPathologyCD10,Pathology.Name,COUNT(patients_affected_by.IdPathologyCD10) FROM patients_affected_by,Pathology WHERE patients_affected_by.IdPathologyCD10=Pathology.IdPathologyCD10 GROUP BY patients_affected_by.IdPathologyCD10 ORDER BY COUNT(patients_affected_by.IdPathologyCD10) DESC;
Result table:

IdPathologyCD10	Name	COUNT
C50	Malignant neoplasm of breast	122
C85	Other specified and unspecified types of non-Hodgkin lymphoma	24
C34	Malignant neoplasm of bronchus and lung	22
C95	Leukemia of unspecified cell type	12
C90	Multiple myeloma and malignant plasma cell neoplasms	11
C16	Malignant neoplasm of stomach	9
C83.3	Diffuse large B-cell lymphoma	8
C61	Malignant neoplasm of prostate	6
C62	Malignant neoplasm of testis	4
C83	Non-follicular lymphoma	4
C19	Malignant neoplasm of rectosigmoid junction	2
C47	Malignant neoplasm of peripheral nerves and autonomic nervous system	2
C57	Malignant neoplasm of other and unspecified female genital organs	1
C26.9	Malignant neoplasm of ill-defined sites within the digestive system	1
C92	Myeloid leukemia	1
C44	Other and unspecified malignant neoplasm of skin	1
C64	Malignant neoplasm of kidney, except renal pelvis	1
C46	Kaposi's sarcoma	1
C73	Malignant neoplasm of thyroid gland	1
D07.3	Carcinoma in situ of other and unspecified female genital organs	1
C81	Hodgkin lymphoma	1
C18	Malignant neoplasm of colon	1
C54.1	Malignant neoplasm of endometrium	1

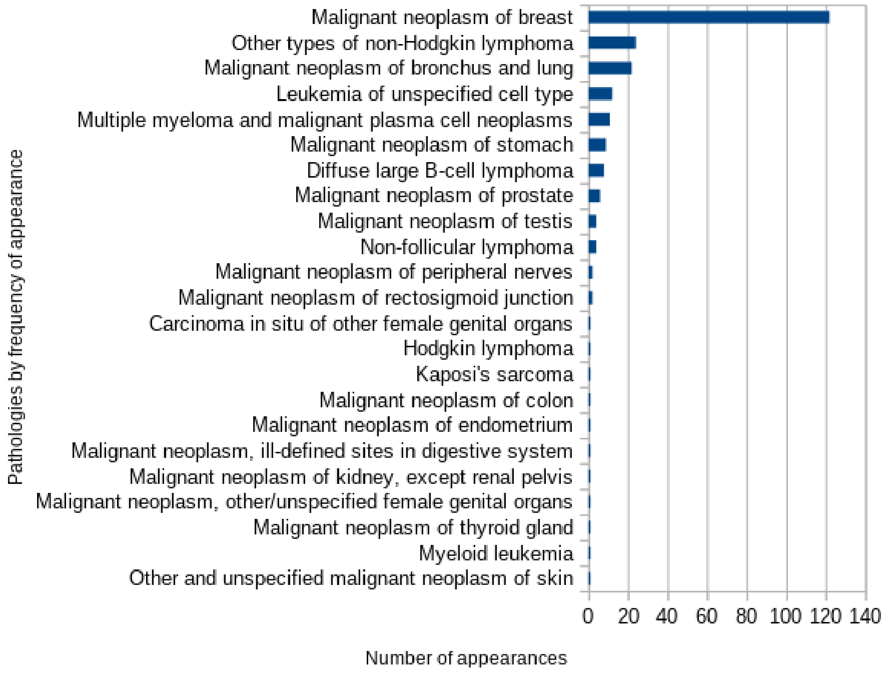

Number of different cardiovascular symptoms which appear in any study

Asked query: SELECT COUNT(DISTINCT IdCardiovascularSymptom) FROM exhibit_symptom;
Result table:

COUNT
42

Number of different cardiovascular symptoms which appear in any study ordered by number of occurrences

Asked query: SELECT IdCardiovascularSymptom,COUNT(IdCardiovascularSymptom) FROM exhibit_symptom GROUP BY IdCardiovascularSymptom ORDER BY COUNT(IdCardiovascularSymptom) DESC;
Result table:

IdCardiovascularSymptom	COUNT
decline in left ventricular ejection fraction (LVEF)	125
heart failure	30
arrhythmia	15
congestive hearth failure	13
congestive heart failure	11

IdCardiovascularSymptom	COUNT
cardiac arrest	7
fatal arrhythmia	7
acute coronary artery syndrome	7
coronary syndrome	7
myocardial injury	5
hypertension	4
atherosclerosis	4
atrial fibrillation	3
systolic dysfunction	3
myocardial fibrosis	2
decline in peak filling rate	2
myocardial infarction	2
cardiac failure	2
tricuspid annular plane systolic excursion (TAPSE) decrease greater or equal than 15%	2
cardiovascular death	2
myocytolysis	2
nonfatal myocardial infarction	2
nonfatal stroke	2
patched myocardial necrosis	2
coronary heart disease	2
cancer therapy-related cardiac dysfunction (CTRCD)	2
myocardial damage	2
target lesion revascularization	1
cardiac biomarkers	1
total cardiovascular events	1
dilated cardiomyopathy	1
abnormal values of parameters of heart and left ventricle	1
change in left ventricular diastolic diameter (LVDD)	1
change in ventricular mass	1
high diastolic and systolic diameter	1
higher systolic diameter	1
arterial hypertension	1
subclinical myocardial damage	1
coronary artery disease	1
left ventricular dysfunction	1
symptomatic congestive hearth failure	1
lower fractional shortening	1

Number of different therapies (considering combined therapies) that appear in any study

Asked query: SELECT COUNT(DISTINCT IdTherapy) FROM cancer_tr_used_therapy;
Result table:

COUNT
7

Number of different therapies (considering combined therapies) that appear, ordered by number of occurrences

Asked query: SELECT IdTherapy,COUNT(Idtherapy) FROM cancer_tr_used_therapy GROUP BY IdTherapy ORDER BY COUNT(IdTherapy) DESC;
Result table:

IdTherapy	COUNT
chemotherapy	144
radiotherapy	39
targeted therapy	33
chemotherapy+radiotherapy	15
chemotherapy+targeted therapy	11
immunotherapy	3
hormonal	1

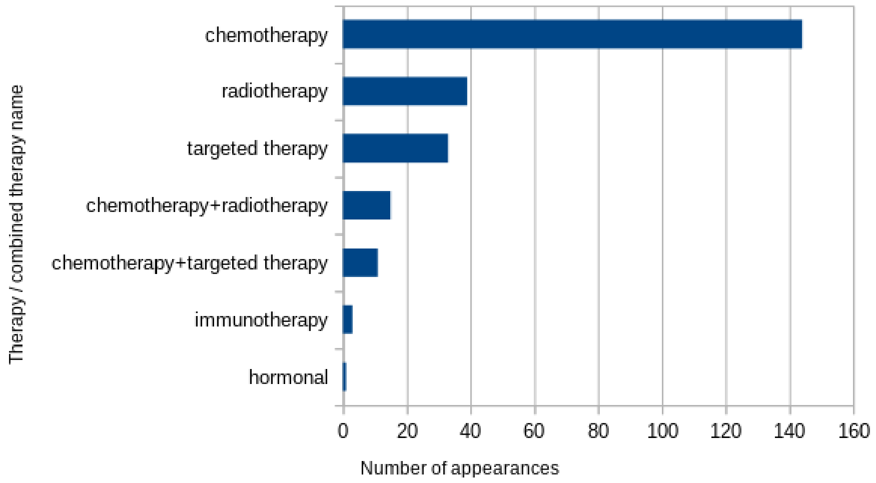

SQL generation and data curation of Oncocardio DB. Page 1 of 3

This is the basic workflow to create the SQL tables for Oncocardio, as long as the SQL sentences to populate them from appropriately curated information.

Directory BASIC_SQL contains the SQL macro for the creation of the tables. This may have been created manually or generated with any program from the entity/relationship diagram. In our case it will be called DBOnco.sql.

The next of the steps described here is done automatically by the script recreate.sh. Nevertheless, it is described here for anyone interested in reproducing the methodology with a different database

From now on, -> is the Unix/Linux prompt so

```
-> ./recreate.sh
```

does everything. Nevertheless, and to explain each step:

0) This script needs to have some software installed. Namely:

  *) R with package stringr and package org.Hs.eg.db from Bioconductor. To install it, from the R prompt do:

```
install.packages(stringr)

if (!require("BiocManager", quietly = TRUE))
    install.packages("BiocManager")
BiocManager::install("org.Hs.eg.db")
```

  *) A recent Perl version with packages perl-List-MoreUtils, perl-Scalar-Util-LooksLikeNumber and perl-Algorithm-Combinatorics

1) Remove all the .sql files in the current folder to be sure all will be reconstructed

```
-> rm -f *.sql
```

2) The file generated automatically by your program to build the SQL script from the table diagrams or E/R diagram may need some minimal corrections related with SQL dialects. In our case this is done by the macro CorrectTable.pl applied as

```
-> ./CorrectTable.pl BASIC_SQL/DBOnco.sql
```

3) Generate the .sql to drop tables (dropall.sql) based on the .sql to create them using the script DB2drop.pl. This script needs the files doall.sql and delete_all_studies.sql

```
-> ./DB2drop.pl DBOnco.sql
```

4) Other two .sql files will be needed. These are static and only have to be copied from the BASIC_SQL folder:

```
cp BASIC_SQL/doall.sql .
cp BASIC_SQL/delete_all_studies.sql .
```

5) Clean all the database and fill the part of it that depends on "static" knowledge. This will be done by the SQL script doall.sql, which:

+ Calls to dropall.sql to erase the relevant tables (if any)
+ Calls to DBOncol.sql to create the empty tables
+ Calls to ten .sql scripts of the form Fill...sql that must be generated. To do so:

5.1) Go to directory EXTRACT and execute ./dogenetables.sh to generate .sql related with tables of genes (Gene, Transcript, Synonim, etc.)

```
-> cd EXTRACT
-> rm -f *.sql
-> R --no-save < dogenetables.R
-> mv -f *.sql ../
-> cd ..
```

Since the information for the gene databases is taken from public databases using R packages, R must be installed in your system including the packages org.Hs.eg.db and stringr (used by the R macro FillTables.R). The first of them, in turn, requires packages AnnotationDbi, stats4, BiocGenerics and parallel which in turn need Biobase, IRanges and S4Vectors.

5.2) Go to directory GENSQL and execute ./dotables.sh to generate .sql related with the tables Drug, Pahtologies and Therapies. They need the text files ATCsimp.csv and icd10cm_order_2021.txt, extracted or downloaded from the corresponding sites, and the perl macros build to generate the correct SQL sentences:

```
-> cd GENSQL
-> rm rm -f *.sql
-> ./FillDrug.pl ATCsimp.csv
-> ./FillTherapy.pl
-> ./FillPathology.pl icd10cm_order_2021.txt
-> ./FillVariationType.pl
-> mv -f *.sql ../
-> cd ..
```

5.3) Fill all tables which contain the actual data from studies by creating the .sql scripts from the .csv file filled manually from the papers describing the studies. This file is data.csv in directory FILL_FROM_CSV

Since most people prefer to work with spreadsheets in Excel format, .xlsx can be used too to contain the information but must be exported to .csv at the end. If you do so from LibreOffice the parameters for .csv export should be:

Character set: Unicode (TUF-8)
Field delimiter: (a blank space)
String delimiter: " (the double quote)
Save cell content as show: UNmarked
Save cell formulas instead of calculated values: UNmarked
Quote all text cells: Marked <--
Fixed column width: UNmarked

The perl macro FILL_FROM_CSV/FillFromPapers.pl will generate the .sql scripts from data.csv. It needs the text file fields.csv to check field names. The rationale for data curation and actual algorithm to generate the .sql sentences are described in file FILL_FROM_CSV/filling_algorithm.txt. Actual generation must be done as:

```
-> cd FILL_FROM_PAPERS
-> ./FillFromPapers.pl data.csv fill_all_studies.sql > inform.txt
-> mv -f *.sql ../
```

This generates a file (inform.txt) which contains two parts: the syntactic analysis (to verify that each field has a legal data type: number, boolean, string..) and that fields with restricted values have legal values (for instance: all pathologies are listed in the ICD10 list, all drugs are included in the ATC list, and so on).

6) Next we need to create the database and introduce all the information in it. According to your configuration you should run MySQL and have access to the database prompt. Assuming you have created a database named oncocardio you should be able to access the SQL console as

```
mysql --silent -u oncocardio -p oncocardio
```

At the SQL prompt execute:

```
MariaDB [oncocardio]> source doall.sql;
MariaDB [oncocardio]> source fill_all_studies.sql;
```

If, later on, you want to keep the static tables and fill only the variable part (studies), for example when you have added more studies, just do

```
MariaDB [oncocardio]> source delete_all_studies.sql;
MariaDB [oncocardio]> source fill_all_studies.sql;
```

Using the OncoCardio database with a different domain

J. Domingo (Juan.Domingo@uv.es)

This guide is intended for system administrators who want to run the oncocardio database with the purpose of reproducibility or to get inspiration to build a similar system with a different database.

The simplest way to test the system is to use it by running the virtual machine we provide. It is a .vmdk disk image file that can be run with VMWare (either Player or Workstation) or with qemu/KVM. From now on, the virtual machine that is run in that way will be called the 'virtual server'.

Step 1 : Choose and register a machine name.

First, you will have to choose a virtual host name (in our case it was 'biodb') so the virtual server will be yourname.your.domain (in our case, biodb.uv.es). Ask your system administrators for an IP to be assigned to it. If you plan to configure the network of the virtual server by DHCP, generate randomly a MAC (but take note of it) and deliver it to your network administrators that will associate it to the new IP.

Step 2 : Prepare network in real host.

Network configuration has been thought to run the virtual server in a real machine with two or more network cards using one of them as a dedicated network interface for the virtual server. This is to prevent conflicts and to assign more easily a different IP number and domain to the virtual server.

The network interface intended to be used for the virtual server must be left untouched by the host (the real machine). To do so:

If you use NetworkManager : prevent NetworkManager from using such card. Directions to do so can be found for example in https://support.qacafe.com/knowledge-base/how-do-i-prevent-network-manager-from-controlling-an-interface/

If you do not use NetworkManager :

a) In Fedora/Rocky Linux/Redhat: do not add a /etc/sysconfig/ifcfg-<interface> for that interface.

b) In Debian/Ubuntu: Alter the /etc/network/interfaces according to the directions in https://wiki.debian.org/NetworkConfiguration

Step 3 : Create a virtual machine.

Now create a virtual machine with VMPlayer or qemu/KVM (with virt-manager). We recommend to assign it either one or two virtual CPUs, and about 2GB of RAM. The operating system obviously must not be installed since it is already installed, together will all the needed software, in the .vmdk image we provide (if VMWare asks for the version, it is a Fedora 32). This virtual disk is to be set as primary (and probably only) disk of the first virtual SATA interface.

The only detail to be specially changed in the creation of the virtual machines is the network configuration:

For creation with virt-manager : set as network source a "Macvtap device" and as device name, the name the host gives to the dedicated network card (in our case, it was `enp3s0` but find it with command `ip link show` or `ifconfig -a`). If you plan to boot the virtual server with dhcp, edit the XML and alter the MAC of this virtual interface to match the one your DHCP server expects. More information on this can be found in https://linuxconfig.org/how-to-use-bridged-networking-with-libvirt-and-kvm

For creation using VMWare : mark the network adapter of the virtual machine as 'Connect at power on' and 'Bridged' and, in the 'Advanced' part of the network configuration, set the MAC to the one expected by your DHCP server if you plan to use dhcp.

Step 4 : Virtual machine boot configuration (change of name and domain).

Now, boot the virtual server. It boots in multi-user mode (runlevel 3, no graphical interface). Log in as `root`, with password `oncocardiopwd`. Change the root password if you wish with command `passwd`.

From now on, all commands must be executed as the superuser (root).

Step 4a : Virtual DHCP network configuration.

The virtual server is currently prepared to boot and start the network using DHCP, so if you have used that and asked for a new IP, the IP should have been assigned as long as the machine name.

Step 4b : Alternative manual network configuration

If you do not use DHCP and prefer to configure the virtual host network manually, execute these commands:

```
cd /etc/sysconfig/network-scripts/
cp ./tmp/ifcfg-ens1 ./
nano ifcfg-ens1
```

This will open an editor to change the network configuration file. Only the values for `HWADDR, IPADDR, NETMASK` and `GATEWAY` should be altered with the correct values that you will have to ask to your network administrator (except the HWADDR, that must be the one you chose as MAC when creating the virtual machine). Finally, edit the file `/etc/hostname` and write there your machine name and domain.

After this, execute

```
systemctl stop NetworkManager
systemctl disable NetworkManager
systemctl restart network
```

Step 5 : Network check.

Whatever the way you have configured the network, you can check that it is working with commands

systemctl status network (which should inform of 'running' status)
systemctl status NetworkManager (as the previous)
ip addr show (which should show the MAC and IP address assigned to the network interface)
hostname (which should show the name of the machine)
hostname -d (which should show the domain of the machine)
hostname -i (which should show the IP address that must coincide with the one shown by the ip command before)

Step 6 : Acquisition of certificates.

To be able to operate in secure mode (encrypted hypertext transfer protocol, or https) you need to obtain the TLS certificates recognized by an external validation authority. Currently, Let's Encrypt (a nonprofit certificate authority ruled by the Internet Security Research Group) provides such certificates for free. Details on the procedure to set up can be found in https://certbot.eff.org/instructions.

Nevertheless, the provided virtual server has already installed the software to get and update such certificates. You only have to execute command

```
certbot certonly --nginx
```

and answer the questions about your server name, domain and organization. This will create the folder /etc/letsencrypt/live/<your-server-name> containing four files (in fact, symbolic links to files with the same name in /etc/letsencrypt/archive/<your-server-name>) named cert.pem, chain.pem, fullchain.pem and privkey.pem.

Step 7 : Setting up the server.

Now, edit the file /etc/nginx/nginx.conf and replace all references to biodb.uv.es by your virtual server name-domain. It appears five times, in lines which currently say

```
server_name biodb.uv.es;
ssl_certificate /etc/letsencrypt/live/biodb.uv.es/fullchain.pem; # managed by Certbot
ssl_certificate_key /etc/letsencrypt/live/biodb.uv.es/privkey.pem; # managed by Certbot
if ($host = biodb.uv.es)
server_name biodb.uv.es;
```

Step 8 : Generate internal certificates.

Use the newly obtained certificates to generate the certificates for the internal tomcat server. To do so, the commands are (substitute yourserver.your.domain by you particular value and oncocardiopwd by any password you choose. We suggest to use the same as for the root password of the virtual server):

```
cd /etc/certs/yourserver.your.domain
openssl pkcs12 -export -in fullchain.pem -inkey privkey.pem -out server.p12 \
-name tomcat
```

```
keytool -importkeystore -deststorepass oncocardiopwd -destkeypass oncocardiopwd \
-destkeystore /etc/tomcat/fkeystore.jks -srckeystore server.p12 \
-srcstoretype PKCS12 -srcstorepass oncocardiopwd -alias tomcat
rm server.p12
```

where the symbol \at the end of a line means 'follows in the same line'.

Step 9 : Update references to new server name.

Some configuration files must be changed to refer to you new host/domain name. In particular:

Substitute the references to `biodb.uv.es` by your server and domain in the following files:

`/home/dist/ocserv.js` (several references)
`/etc/tomcat/server.xml` (just one reference in line `keystoreFile="/etc/tomcat/biodb.jks"`)

and change appropriately according to your preferences the file

`/etc/oncocardio/mailtext`

which is the text sent by mail to the user when they receive their results attached.
Also, change and the file

`background.png`

which is the background the users see in the oncocardio screen.

Step 10 : Reboot

Just reboot the virtual machine with command `reboot`. Then you should be able to use a browser to access by https to your domain. The oncocardio database should work in your domain.
