# Supplementary material for: OncoCardioDB: a public and curated database of molecular information in onco-cardiology/cardio-oncology
Source: Database (Oxford). 2023 May 9;2023:baad029. doi: 10.1093/database/baad029 (PMC10167979; doi:10.1093/database/baad029)
Supplement: baad029_Supp [file baad029_supp.zip › suppl_data/OncoCardioSup4B.tgz!/DBOncoClean/README.pdf]

SQL generation and data curation of Oncocardio DB. Page 1 of 3

This is the basic workflow to create the SQL tables for Oncocardio, as long as the SQL sentences to populate them from appropriately curated information.

Directory BASIC_SQL contains the SQL macro for the creation of the tables. This may have been created manually or generated with any program from the entity/relationship diagram. In our case it will be called DBOnco.sql.

The next of the steps described here is done automatically by the script recreate.sh. Nevertheless, it is described here for anyone interested in reproducing the methodology with a different database

From now on, -> is the Unix/Linux prompt so

```
-> ./recreate.sh
```

does everything. Nevertheless, and to explain each step:

0) This script needs to have some software installed. Namely:

*) R with package stringr and package org.Hs.eg.db from Bioconductor. To install it, from the R prompt do:

```
install.packages(stringr)

if (!require("BiocManager", quietly = TRUE))
    install.packages("BiocManager")
BiocManager::install("org.Hs.eg.db")
```

*) A recent Perl version with packages perl-List-MoreUtils, perl-Scalar-Util-LooksLikeNumber and perl-Algorithm-Combinatorics

1) Remove all the .sql files in the current folder to be sure all will be reconstructed

```
-> rm -f *.sql
```

2) The file generated automatically by your program to build the SQL script from the table diagrams or E/R diagram may need some minimal corrections related with SQL dialects. In our case this is done by the macro CorrectTable.pl applied as

```
-> ./CorrectTable.pl BASIC_SQL/DBOnco.sql
```

3) Generate the .sql to drop tables (dropall.sql) based on the .sql to create them using the script DB2drop.pl. This script needs the files doall.sql and delete_all_studies.sql

```
-> ./DB2drop.pl DBOnco.sql
```

4) Other two .sql files will be needed. These are static and only have to be copied from the BASIC_SQL folder:

```
cp BASIC_SQL/doall.sql .
cp BASIC_SQL/delete_all_studies.sql .
```

5) Clean all the database and fill the part of it that depends on "static" knowledge. This will be done by the SQL script doall.sql, which:

+ Calls to dropall.sql to erase the relevant tables (if any)
+ Calls to DBOncol.sql to create the empty tables
+ Calls to ten .sql scripts of the form Fill...sql that must be generated. To do so:

5.1) Go to directory EXTRACT and execute ./dogenetables.sh to generate .sql related with tables of genes (Gene, Transcript, Synonim, etc.)

```
-> cd EXTRACT
-> rm -f *.sql
-> R --no-save < dogenetables.R
-> mv -f *.sql ../
-> cd ..
```

Since the information for the gene databases is taken from public databases using R packages, R must be installed in your system including the packages org.Hs.eg.db and stringr (used by the R macro FillTables.R). The first of them, in turn, requires packages AnnotationDbi, stats4, BiocGenerics and parallel which in turn need Biobase, IRanges and S4Vectors.

5.2) Go to directory GENSQL and execute ./dotables.sh to generate .sql related with the tables Drug, Pahtologies and Therapies. They need the text files ATCsimp.csv and icd10cm_order_2021.txt, extracted or downloaded from the corresponding sites, and the perl macros build to generate the correct SQL sentences:

```
-> cd GENSQL
-> rm rm -f *.sql
-> ./FillDrug.pl ATCsimp.csv
-> ./FillTherapy.pl
-> ./FillPathology.pl icd10cm_order_2021.txt
-> ./FillVariationType.pl
-> mv -f *.sql ../
-> cd ..
```

5.3) Fill all tables which contain the actual data from studies by creating the .sql scripts from the .csv file filled manually from the papers describing the studies. This file is data.csv in directory FILL_FROM_CSV

Since most people prefer to work with spreadsheets in Excel format, .xlsx can be used too to contain the information but must be exported to .csv at the end. If you do so from LibreOffice the parameters for .csv export should be:

Character set: Unicode (TUF-8)
Field delimiter: (a blank space)
String delimiter: " (the double quote)
Save cell content as show: UNmarked
Save cell formulas instead of calculated values: UNmarked
Quote all text cells: Marked <--
Fixed column width: UNmarked

The perl macro FILL_FROM_CSV/FillFromPapers.pl will generate the .sql scripts from data.csv. It needs the text file fields.csv to check field names. The rationale for data curation and actual algorithm to generate the .sql sentences are described in file FILL_FROM_CSV/filling_algorithm.txt. Actual generation must be done as:

```
-> cd FILL_FROM_PAPERS
-> ./FillFromPapers.pl data.csv fill_all_studies.sql > inform.txt
-> mv -f *.sql ../
```

This generates a file (inform.txt) which contains two parts: the syntactic analysis (to verify that each field has a legal data type: number, boolean, string..) and that fields with restricted values have legal values (for instance: all pathologies are listed in the ICD10 list, all drugs are included in the ATC list, and so on).

6) Next we need to create the database and introduce all the information in it. According to your configuration you should run MySQL and have access to the database prompt. Assuming you have created a database named oncocardio you should be able to access the SQL console as

```
mysql --silent -u oncocardio -p oncocardio
```

SQL generation and data curation of Oncocardio DB. Page 3 of 3

At the SQL prompt execute:

```
MariaDB [oncocardio]> source doall.sql;
MariaDB [oncocardio]> source fill_all_studies.sql;
```

If, later on, you want to keep the static tables and fill only the variable part (studies), for example when you have added more studies, just do

```
MariaDB [oncocardio]> source delete_all_studies.sql;
MariaDB [oncocardio]> source fill_all_studies.sql;
```
